# Effects of pharmacists’ involvement in pharmacogenomics implementation on patient outcomes: a scoping review

**DOI:** 10.1080/20523211.2026.2746884

**Published:** 2026-11-03

**Authors:** Nadia Al Mazrouei, Khalid Awad Al-Kubaisi, Karem H. Alzoubi, Ahmed Alhusban, Richard Mottershead, Moh'D. Ahmad Shara, Manal Mohammed Al Sharawy, Margaret Stevenson

**Affiliations:** a Department of Pharmacy Practice and Pharmacotherapeutics, College of Pharmacy, University of Sharjah, Sharjah, United Arab Emirates; b Department of Clinical Pharmacy, Faculty of Pharmacy, Jordan University of Science and Technology, Irbid, Jordan; c Department of Medical Education, College of Medicine and Health Sciences, United Arab Emirates University, Al Ain, United Arab Emirates; d College of Nursing, University of Baghdad, Baghdad, Iraq; e Department of Pharmacy Practice and Pharmacotherapy, College of Pharmacy, University of Sharjah, Sharjah, United Arab Emirates; f Department of Laboratory Medicine and Pathobiology, University of Toronto, Toronto, Canada

**Keywords:** PGx, pharmacists, clinical workflow, personalised medicine, genomics implementation, patient care

## Abstract

**Background:**

Pharmacogenomics holds promise for personalised medicine, but its implementation requires effective integration into clinical workflows. Pharmacists, with their medication expertise, are positioned to play a key role in implementing these services. However, a comprehensive synthesis of evidence regarding pharmacists’ roles in pharmacogenomic implementation has been lacking.

**Methods:**

A scoping review was conducted following a pre-registered protocol and PRISMA-ScR guidance. PubMed/MEDLINE, Scopus, Cochrane Library, CINAHL, and PsycINFO were searched from January 2015 to September 2026, supplemented by grey literature and reference screening. Studies examining pharmacist involvement in clinical PGx implementation were eligible. Data were extracted, critically appraised using design-specific tools, and synthesised thematically.

**Results:**

From an initial yield of 870 records identified through database and grey literature searching, studies were screened, and eligible evidence was synthesised in this scoping review. Included literature comprised cross-sectional (n = 2), quasi-experimental (n = 1), case series (n = 5), qualitative (n = 1), mixed-methods (n = 1), descriptive implementation (n = 3), and an AI-based clinical decision-support evaluation (n = 1). Overall methodological quality was moderate to low, reflecting predominantly observational and implementation-oriented designs. Thematic synthesis identified key domains: pharmacist roles in PGx workflow integration, result interpretation, clinical decision support use, interprofessional collaboration, patient education, medication optimization, implementation leadership, and system-level barriers.

**Conclusion:**

Pharmacists are central to successful PGx implementation across diverse healthcare settings. Although evidence supports improvements in implementation processes and prescribing decisions, robust evidence demonstrating long-term clinical and economic benefits remains limited. Future research should prioritise rigorous outcome-focused evaluations to support sustainable integration of pharmacist-led PGx services.

## Background

The landscape of modern healthcare is increasingly shaped by personalised medicine, providing a new frontier in prevention and treatment pathways based on individual patient characteristics (Abad-Santos et al., 2024; Amaro-Álvarez et al., 2024; Liang et al., 2025; Maltsev et al., 2025; Sattar et al., 2023; Showbharnikhaa et al., 2024; Silva et al., 2024; Stratton & Olson, 2023; Taherdoost & Ghofrani, 2024; White et al., 2022). Pharmacogenomics (PGx) is an important component of this approach, using genomic information to account for variation in drug response and inform individualised drug selection and dosing (Kabbani et al., 2023; Royal College of Pharmacy, 2025). The clinical relevance of PGx has been demonstrated across cardiovascular medicine, oncology and psychiatry, where genetic variation can influence treatment response and medication safety (Alchakee et al., 2022; Marcu & Marcu, 2024; Ross et al., 2023). Pharmacists are increasingly recognised as potential facilitators of PGx implementation because of their expertise in pharmacotherapy and medication management, with reported roles encompassing genetic result interpretation, medication therapy management, patient education and counselling, prescriber collaboration, clinical decision-support development, and programme management (Alchakee et al., 2022; Alhaddad et al., 2022; Gammal et al., 2022; Gammal & Fieg, 2022; Hu et al., 2022; Odeh et al., 2024; Ross et al., 2023).

Despite this potential, important gaps remain in the implementation and evaluation of pharmacist-led PGx services. Pharmacist expertise and accessibility may support safer prescribing through identification of clinically relevant drug–gene interactions and optimization of drug selection (Al Maruf & Aziz, 2023; Rutkowska et al., 2026), although reported benefits, including reductions in adverse drug events and improvements in medication adherence, require cautious interpretation given the variability of the underlying evidence (Chenchula et al., 2024). Existing literature identifies limitations in pharmacist education and training, variability in implementation models, electronic health record (EHR) and clinical decision-support integration, reimbursement, fragmented care pathways, and evidence on patient outcomes and healthcare costs (Bain et al., 2020; Cohen et al., 2013; Mai et al., 2024; Qureshi et al., 2022; Shue et al., 2024; Smith et al., 2025; Virelli et al., 2021). Knowledge and attitude reviews indicate strong support for PGx among pharmacists and pharmacy students but inconsistent knowledge and confidence, suggesting a workforce-readiness gap (Hansen et al., 2022; Wondrasek et al., 2024). Similarly, implementation reviews demonstrate feasibility across primary care, hospital, and community settings but substantial heterogeneity, limited standardization, and dependence on infrastructure maturity (Bain et al., 2018; Hayward et al., 2021; Wu et al., 2025). Reviews of precision medicine further position pharmacists as important contributors to biomarker-guided therapy, particularly in oncology, although much of this literature remains conceptual rather than evaluative (Hayashi et al., 2022; Moore & Guinigundo, 2023).

Considering these aspects, it can be argued that while the literature suggests that pharmacist involvement in PGx is feasible, the extent to which implementation translates into measurable clinical benefit remains insufficiently established. Recurrent barriers include EHR integration, clinical decision-support limitations, reimbursement and fragmented care pathways (Bain et al., 2018; Hayward et al., 2021). Concomitantly, the evidence base remains dominated by feasibility studies, surveys and descriptive implementation reports, with limited assessment of methodological robustness and downstream outcomes. Therefore, this scoping review aims to address these gaps by systematically mapping and synthesizing the existing evidence on pharmacists’ roles in implementing PGx within personalised medicine. Specifically, we will explore the current landscape of pharmacist-led PGx applications, identify implementation strategies and barriers, and synthesise evidence on the impact of pharmacist involvement on treatment outcomes and patient care.

## Methods

### Protocol registration

This scoping review was conducted following a pre-specified protocol registered with the Open Science Framework (OSF). The review methodology adhered to the PRISMA-ScR guidelines for scoping reviews, with the complete checklist available in Supplemental Material 1.

### Search strategy and information sources

#### Search strategy

A comprehensive and systematic search was conducted in accordance with the Joanna Briggs Institute (JBI) methodology (Aromataris et al., 2024) for scoping reviews and PRISMA-ScR reporting guidelines (Tricco et al., 2018). Electronic databases searched included PubMed/MEDLINE, Scopus, Cochrane Library, CINAHL, and PsycINFO from January 2015 to September 2026. The search strategy was developed iteratively in consultation with a health sciences librarian and combined controlled vocabulary (e.g. MeSH terms) with free-text keywords to ensure sensitivity for pharmacist-led PGx implementation literature. The search string used is provided in Supplemental Material 2. The final search strategy was structured around three core conceptual domains: (1) PGx, (2) pharmacist involvement, and (3) implementation or clinical integration.

Database-specific adaptations were applied for controlled vocabulary indexing terms. Filters were applied to exclude secondary research, such as systematic and narrative reviews, to avoid duplication of evidence and ensure that the review focused on eligible primary research and relevant expert-opinion literature. In addition to peer-reviewed databases, grey literature was searched using Google Scholar (first 200 results sorted by relevance), professional society websites (e.g. CPIC, ASHP), and conference abstracts where implementation of PGx services by pharmacists was described. Reference lists of included studies were manually screened for additional eligible records.

#### Eligibility criteria

Studies were eligible for inclusion if they examined pharmacist involvement in PGx within clinical practice. The population of interest comprised pharmacists or pharmacy services actively engaged in the implementation of PGx approaches across healthcare settings. These included roles spanning testing initiation, result interpretation, clinical decision support (CDC), and integration of PGx data into routine medication management workflows.

The conceptual focus of eligible studies was PGx-related clinical service delivery. This encompassed a broad range of implementation activities, including but not limited to genetic testing services, interpretation of gene-drug interactions, use of clinical CDC systems, integration of PGx data into prescribing workflows, and pharmacist-led interventions aimed at optimizing pharmacotherapy based on genetic information. The context of inclusion was intentionally broad to capture real-world implementation across diverse healthcare environments. Eligible studies could be conducted in any clinical setting, including outpatient clinics, oncology services, veterans’ health systems, community pharmacies, primary care environments, and retirement or long-term care settings, provided that PGx services were operationally implemented within those settings.

In terms of evidence type, the review included primary empirical research designs such as quantitative, qualitative, mixed-methods, quasi-experimental studies, case series, and implementation studies. In addition, structured implementation reports describing real-world service delivery models were eligible for inclusion when they provided substantive methodological detail relevant to service design or workflow integration. Expert opinion and commentary articles were considered eligible when they provided a clearly described PGx implementation framework, service model, workflow, or operational approach relevant to the review question, particularly where comparable empirical evidence was limited. Eligibility was assessed against these predefined criteria by two reviewers independently. Reviewers considered whether the article provided sufficient detail on the implementation approach, pharmacist role, or service delivery model, and its relevance to clinical PGx implementation. Disagreements regarding eligibility were resolved through discussion and consensus between the reviewers. Articles that provided only general commentary, theoretical discussion, or opinions without a clearly defined implementation or operational component were excluded. This approach was used to minimise subjective selection and ensure that expert opinion literature was included only where it contributed substantive implementation evidence. All included studies were required to be published in English between January 2015 and September 2026. This timeframe was selected to capture contemporary developments in clinical PGx implementation, reflecting the period during which PGx services transitioned from research-oriented applications toward routine clinical integration.

#### Study selection

All identified records were imported into Covidence for deduplication and screening. Two independent reviewers screened titles and abstracts. Studies that met the eligibility criteria were retrieved for full-text assessment. Studies were included if they met all of the following criteria. First, they examined the role of pharmacists in the implementation or delivery of PGx (PGx) services. Second, they described at least one pharmacist-related PGx activity, including PGx result interpretation, CDC, medication therapy management, patient or clinician education, testing coordination, workflow integration, or implementation leadership. Third, sufficient methodological or implementation detail was available to permit data extraction. Eligible studies comprised primary research and selected expert-opinion or implementation articles that provided a clearly described PGx service model, workflow, or implementation approach. Secondary research, including systematic, scoping, and narrative reviews, was excluded from the evidence synthesis, although relevant reviews were used for background and reference-list screening. Any disagreements between reviewers were resolved through consensus or third-party adjudication.

### Data extraction and analysis

Data extraction was conducted using a tailored data extraction form in line with the Cochrane Manual for systematic reviews for interventions (Higgins et al., 2024) to ensure consistency and comprehensiveness across all included studies. The extracted variables included study characteristics, such as design and setting, country, funding, initial sample, final sample, demographics, and clinical status, and study-specific notes. Another data summary table was developed in alignment with the research questions set for this scoping review. This included pharmacist roles and responsibilities within PGx services, limitations, and the type of PGx intervention implemented. Particular attention was given to how PGx services were operationalised, including implementation models such as workflow integration, CDS incorporation, and service delivery structures. Data extraction Tables are presented in Supplemental Material 3. In addition, data were collected on reported outcomes, encompassing clinical outcomes, operational metrics, behavioural changes, and broader implementation-related endpoints. Information on barriers and facilitators to implementation was systematically extracted to capture determinants influencing the adoption and sustainability of pharmacist-led PGx services. Contextual health system factors, including institutional characteristics and care delivery environments, were also recorded to support the interpretation of variability across settings.

Quality appraisal was conducted using methodological tools appropriate to each study design. The Joanna Briggs Institute's (Aromataris et al., 2024) critical appraisal tools were applied to case series, cross-sectional, quasi-experimental, and qualitative studies. The Mixed Methods Appraisal Tool (MMAT) (Hong et al., 2018) was used for studies employing mixed-methods designs. For studies involving computational or artificial intelligence-based approaches, the PROBAST + AI framework (BMJ, 2025a) was applied where relevant. In addition, GRADE principles (BMJ, 2025b) were used in a descriptive manner to assess the overall certainty of evidence across outcome domains, particularly in relation to implementation effectiveness and clinical impact. Quality appraisals are presented in Supplemental Material 4. GRADE evaluation for the studies included in this review is presented in Supplemental Material 5.

Data synthesis followed a thematic approach based on a previously described (Thomas & Harden, 2008) methodologies. This involved systematic line-by-line coding of extracted data, the development of descriptive themes, and the generation of higher-order analytical themes. These analytical themes were explicitly aligned with the aim, focusing on pharmacist roles in PGx implementation, the impact on patient care and treatment outcomes, and the key barriers and facilitators influencing integration into clinical workflows. These data are presented in Supplemental Material 6.

## Results

### Search results

A total of 870 records were identified through the database searches and other sources. After the removal of duplicates, the remaining 97 records were scanned via title reads, and another 60 records were removed for misalignment with the inclusion criteria. The remaining 37 records were screened via abstract reads for compatibility with the inclusion criteria. After screening, 18 records were excluded because they did not meet the predefined eligibility criteria. The remaining 19 articles were retrieved for full-text assessment. Following full-text review, 5 articles were excluded for two main reasons: no true PGx implementation or evaluation (n = 3), and study protocols (n = 2). Ultimately, 14 studies met the predefined inclusion criteria and were included in the final synthesis. A PRISMA flow diagram illustrates the study selection process (Figure 1).

**Figure 1.**
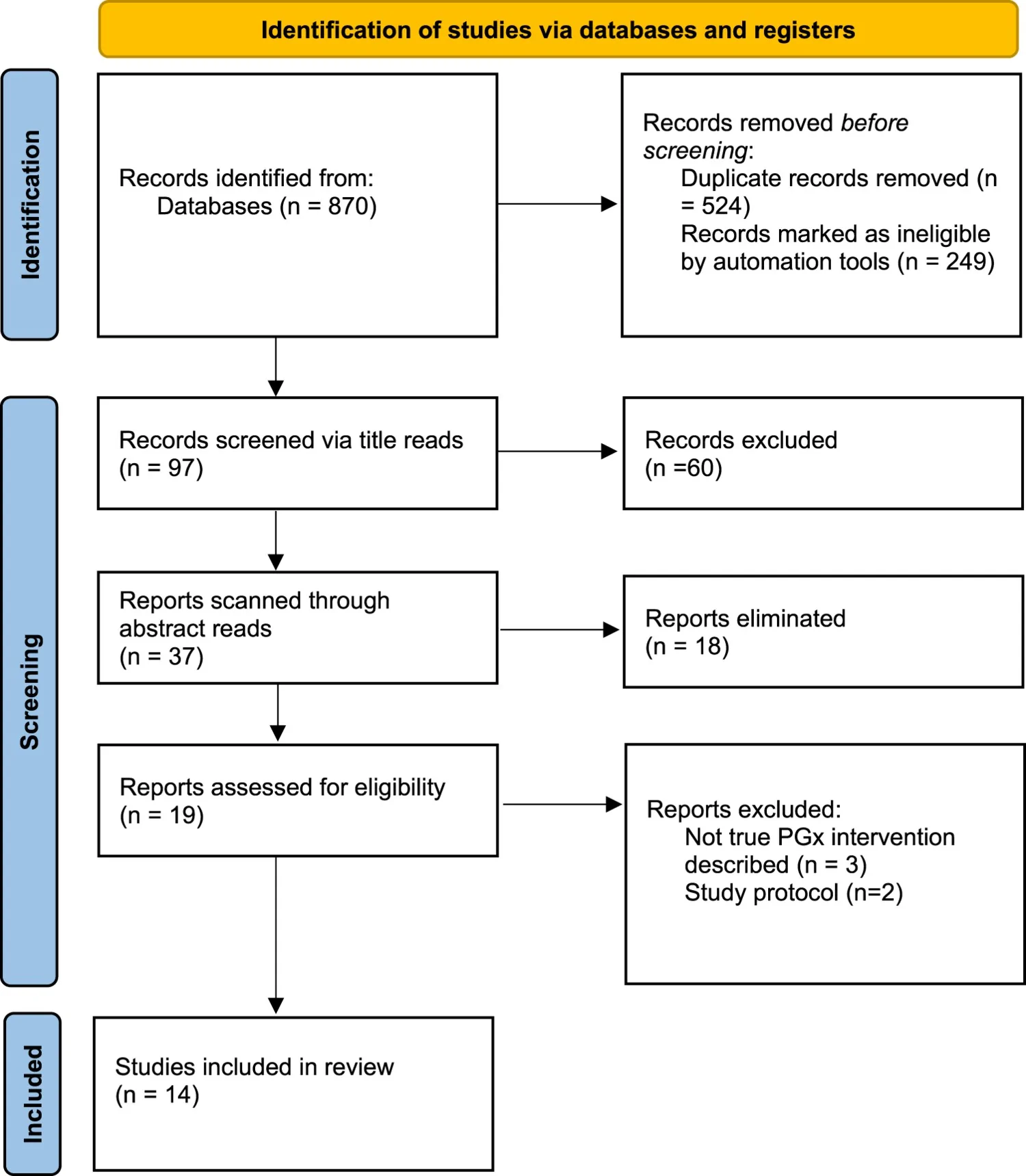
PRISMA flow chart & study selection process.

The evidence was predominantly from the US (Bain et al., 2018; Bain et al., 2019; Bright & Klepser, 2018; Chaitesipaseut et al., 2022; Dreischmeier et al., 2024; Hicks et al., 2016; Liko et al., 2021; McQuillan et al., 2026; Przybylski et al., 2020; Van Heukelom et al., 2023; Wang et al., 2023), with additional studies from Switzerland Jeiziner et al., 2023; and the Netherlands van der Wouden et al., 2020; across community (Bain et al., 2018; Bain et al., 2019; Chaitesipaseut et al., 2022; van der Wouden et al., 2020; Wang et al., 2023) primary care (Liko et al., 2021; van der Wouden et al., 2020) oncology (Przybylski et al., 2020), geriatric (Bain et al., 2019), Veterans Affairs (Dreischmeier et al., 2024), executive health (Liko et al., 2021), and integrated health-system settings (McQuillan et al., 2026). Study designs were heterogeneous, but most were descriptive or observational. Pharmacists contributed to PGx interpretation, medication optimization, counselling, CDC, testing coordination, workflow development, and programme leadership. Thus, evidence was stronger for feasibility and pharmacist roles than for causal clinical effects. The predominance of US-based studies may limit generalisability because pharmacist education, scope of practice, PGx training, prescribing authority, reimbursement, laboratory access, and health-system infrastructure vary across countries.

Critical appraisal identified limitations including non-comparative designs, sampling bias, limited control of confounding, descriptive or self-reported outcomes, and limited external validation of emerging technologies (Supplemental Material 4). GRADE certainty was low for pharmacist roles and feasibility and for clinical outcomes, and moderate for barriers and facilitators (Supplemental Material 5). Overall, the evidence supports the feasibility and defined role of pharmacists in PGx implementation, but provides limited certainty regarding clinical effectiveness. Table 1 provides an overview of studies included for review.

**Table 1.** Overview of included studies examining pharmacist involvement in PGx implementation.

Study	Country	Design/population	Main findings	Pharmacist contribution	Barriers/implementation challenges	Evidence profile
Wang et al., 2023	USA	Retrospective chart review; 137 patients, 87 analyzed	Provider adoption 88.5%; substantial reduction in moderate/significant gene–drug interactions; PHQ-9 improved	PGx implementation, counselling, interpretation, recommendations, education, follow-up	Retrospective design; attrition; single-center setting; diagnostic misclassification; pharmacist effect not independently measurable	Observational; non-comparative
Bright & Klepser, 2018	USA	Simulated feasibility study; 54 encounters; 5 pharmacists	PGx point-of-care testing was feasible; pharmacist time reduced when technicians supported testing	Eligibility assessment, consent, sample collection, interpretation, recommendations, prescriber communication, counselling	Need for technician support and workflow delegation; limited PGx scope; simulated rather than real-world implementation	Feasibility; simulated
Jeiziner et al., 2023	Switzerland	Mixed methods; 25 and 42 pharmacy participants	Recommendations implemented in 69%; patients demonstrated good understanding; 62% willing to pay	Testing, counselling, interpretation, MTM, traffic-light medication lists, physician communication	Patient willingness to pay; single-site implementation; limited integration with wider care pathways; selection and recall bias	Mixed-methods; non-comparative
Van Heukelom et al., 2023	USA	Descriptive implementation; 102 patients, 131 visits	77% of referrals were from providers; 64 pre-test visits resulted in 49 (77%) PGx testing orders	Medication review, counselling, testing, interpretation, recommendations, communication, triage, clinician education	Early-stage implementation; small cohort; dependence on provider referrals; single health system; pharmacist effect not isolated	Descriptive implementation
Hicks et al., 2016	USA	Descriptive implementation report	211 PGx results in first 3 months; 17 actionable findings; approximately 2,000 tests/year	EHR/CDS development, result review, interpretation, consultation, education, PGx clinic	Resource-intensive implementation; need for EHR infrastructure and CDS; limited long-term outcome data	Descriptive implementation
van der Wouden et al., 2020	Netherlands	15 semi-structured interviews with community pharmacists; 92 recommendations	Pharmacists viewed themselves as leaders; physician discussion occurred for 77.2% of recommendations	Patient identification, testing, CDS use, interpretation, physician communication, follow-up	Limited pharmacist training/experience; need for physician collaboration; workflow and implementation demands; research-supported setting	Qulaitative
Murugan et al., 2024	USA*	AI development/evaluation; 80 PGx questions; 4 experts	RAG GPT-4 achieved higher provider-focused performance than ChatGPT-3.5	PGx expertise, knowledge-base development, question design, expert evaluation	Retrieval/terminology limitations; complex patient language; absence of external validation; unresolved regulatory, privacy and safety issues	AI development; high evaluation risk of bias
Chaitesipaseut et al., 2022	USA	Prospective single-arm feasibility case series; 18 residents	23 qualifying medication results; 7 (30%) required caution/alternative; one medication change	Testing, interpretation, PGx-MTM, identification of drug-related problems	Small sample; single site; feasibility design; limited evidence of downstream prescribing change	Feasibility case series
Bain et al., 2019	USA	Observational/descriptive case series; 100 PACE participants	169 DGIs, 125 DDGIs and 280 phenoconversions identified	PGx interpretation, CDSS use, DGI identification, clinical support	Dependence on CDSS and available clinical data; limited assessment of prescribing changes; no patient-centered outcomes	Descriptive case series
Bain et al., 2018	USA	Feasibility implementation; 296 PACE participants	73.6% exposed to multiple interactions; 89% of recommendations accepted	PGx interpretation, DGI identification, recommendations, prescriber communication, education	EHR interoperability; limited PGx visibility; restricted/incomplete clinical data; variable feedback; knowledge gaps	Feasibility implementation
Przybylski et al., 2020	USA	Cross-sectional survey; 84 oncology pharmacy professionals; 58 centres	PGx involvement in policy, tumor boards and education associated with greater self-reported comfort	PGx interpretation, policy development, CDS, tumor boards, education	Limited EMR visibility; resource constraints; knowledge and confidence gaps; convenience sampling	Cross-sectional; self-reported
Dreischmeier et al., 2024	USA	Descriptive implementation	PGx workflow successfully embedded within oncology practice	Workflow design, patient selection, testing, interpretation, MTM, documentation, communication	Workflow integration demands; need for dedicated expertise and infrastructure; descriptive evidence without comparator	Implementation report
Liko et al., 2021	USA	Descriptive implementation/expert opinion	Structured multidisciplinary PGx service feasible within executive health	Pre/post-test counselling, medication review, testing, interpretation, therapeutic planning, physician collaboration	Evidence gaps for some gene–drug pairs; communication; scheduling; reimbursement	Expert opinion/implementation
McQuillan et al., 2026	USA	Descriptive early implementation/pilot; VHA; 6 CPhT positions	CPhT model reduced pharmacist administrative workload and improved timeliness/reliability of PGx processes	Workforce design, CPhT training, pharmacist oversight, triage, clinical review, workflow integration	Training requirements; infrastructure needs; need for pharmacist oversight; scalability and generalisability; contribution of pharmacist vs CPhT not independently quantified	Early implementation report

Note: drug-gene interactions (DGIs); drug-drug-gene interactions (DDGIs); medication therapy management (MTM); point-of-care testing (POCT); clinical pharmacy technician (CPhT). Formal GRADE ratings are reported in Supplemental Material 5; detailed critical appraisal is provided in Supplemental Material 4.

### Thematic analysis

The thematic analysis identified four overarching themes (Clinical Translation of PGx Information; Patient and Clinician Education and Capacity Building; Program Management and Health-System Integration; PGx Research, Evidence Generation, and Emerging Technologies) describing pharmacist involvement in PGx implementation. Details are provided in Supplemental Material 6.

### Clinical translation of PGx information

#### Interpretation and CDC

Interpretation of PGx results was a central pharmacist function across the included implementation models. Pharmacists translated laboratory-generated genetic information into clinically actionable recommendations by integrating genotype with the patient's medication profile, clinical indication, and relevant drug–gene interactions. This function was demonstrated across primary care, community pharmacy, geriatric care, oncology, and integrated health-system settings (Bain et al., 2018, 2019; Bright & Klepser, 2018; Dreischmeier et al., 2024; Hicks et al., 2016; Jeiziner et al., 2023; van der Wouden et al., 2020; Wang et al., 2023).

In the community-based behavioural health study (Wang et al., 2023), pharmacists interpreted GeneSight® results and translated PGx classifications into psychotropic treatment recommendations. Following PGx-guided medication modification, prescribing shifted significantly toward medications without identified gene–drug interactions, increasing from 44.0% to 87.4%, while medications associated with moderate and significant interactions decreased from 35.2% to 7.9% and 11.2% to 2.1%, respectively. Depression severity also decreased significantly, with mean PHQ-9 scores falling from 16.1 to 11.2. However, these findings cannot establish that pharmacist involvement caused the clinical improvement because the study was a retrospective, single-center analysis without a control group, and the pharmacist intervention was embedded within a multidisciplinary service. The clinical outcome was also available for only 59 patients, further reducing certainty.

In geriatric populations (Bain et al., 2019) pharmacists addressed a substantially more complex interpretive problem. The investigation identified 169 drug–gene interactions, 125 drug–drug–gene interactions, and 280 phenoconversion events among 100 PACE participants. These findings demonstrate the potential value of pharmacist interpretation in populations in which genotype cannot be considered independently of polypharmacy and concurrent drug exposure. Nevertheless, the observational design did not establish whether identification of these interactions resulted in clinically meaningful changes in therapy or patient outcomes.

CDS systems provided an important mechanism for translating genomic information into prescribing decisions. In this sense, an investigation (Hicks et al., 2016) tested 63 PGx integrated alerts and rules in the EHR and combined automated alerts with pharmacist review. During the initial implementation period, pharmacists identified and acted upon 17 actionable PGx findings among 211 results. This model illustrates the complementary relationship between automation and professional judgement: CDS facilitated identification of relevant findings, whereas pharmacists adjudicated results and addressed situations not adequately captured by automated rules. Similar integration was reported by others (Bain et al., 2019; Bright & Klepser, 2018; Van Heukelom et al., 2023). Nonetheless, a major limitation was the dependence of CDS effectiveness on the quality and accessibility of the underlying clinical infrastructure. Incomplete EHR interoperability, restricted clinical information, variability in decision-support functionality, and the need for manual review constrained implementation. Thus, CDS should not be interpreted as a standalone intervention, as its clinical value may be linked with integration capacity, pharmacist expertise, and established prescribing workflows.

#### Provider consultation and interprofessional translation

Pharmacists also served as an intermediary between PGx evidence and prescribing clinicians. This involved communicating recommendations, responding to PGx-related clinical questions, and supporting physicians and other prescribers in applying genetic information to individual treatment decisions. One investigation (Van Heukelom et al., 2023) reported that pharmacists discussed PGx recommendations with physicians in 77.2% of cases, while another study (van der Wouden et al., 2020) demonstrated that recommendations could be communicated to both referring and non-referring physicians. In one study (Wang et al., 2023), medication treatment was consistent with PGx recommendations in 88.5% of patients.

Evidence from oncology further indicates that pharmacist participation may contribute to institutional readiness. In this respect, an investigation (Przybylski et al., 2020) found that involvement in PGx policy development, molecular tumor boards, and PGx education was associated with greater self-reported comfort in interpreting PGx information among oncology pharmacy professionals. However, this was a cross-sectional survey based on self-reported comfort rather than objectively assessed competence or prescribing behaviour. This limitation is also observed in other studies (Bain et al., 2019; Bright & Klepser, 2018; Chaitesipaseut et al., 2022; Dreischmeier et al., 2024; Hicks et al., 2016; Jeiziner et al., 2023; Liko et al., 2021; McQuillan et al., 2026; Murugan et al., 2024; Przybylski et al., 2020; van der Wouden et al., 2020; Van Heukelom et al., 2023; Wang et al., 2023) issuing recommendations for uptake despite absent data on improved patient outcomes.

#### MTM and clinical optimization

In pharmacist-led interventions for PGx-informed MTM, pharmacists converted genomic findings into changes in pharmacotherapy with overall positive outcomes for patients. Notably, one study (Chaitesipaseut et al., 2022) demonstrated the feasibility of pharmacist-led PGx-MTM in a retirement community, where 7 of 23 qualifying medication results required caution or consideration of an alternative therapy, and one participant underwent an actual medication change. Other research similarly reported a high burden of clinically relevant interactions (Bain et al., 2019), while the earlier implementation study reported acceptance of 89.0% of pharmacist recommendations (Bain et al., 2018)

These findings support the operational feasibility of incorporating PGx into medication review, particularly in populations with polypharmacy and multiple potential gene–drug interactions. However, the evidence remains insufficient to determine whether identifying and modifying pharmacotherapy based on PGx consistently reduces adverse drug reactions, hospitalization, or other patient-centered outcomes. Most studies were single-arm or observational, and the definitions of prescribing improvement were heterogeneous. The evidence therefore supports clinical optimization as an established pharmacist activity, but not yet as a definitively effective intervention for improving long-term clinical outcomes.

### Patient and clinician education and capacity building

Research on education and capacity building for PGx was reported in the literature at two distinct levels. Firstly, pharmacists supported patients in understanding and participating in PGx-guided care, and secondly, pharmacists supported clinicians in developing the knowledge required to use PGx information appropriately.

#### Patient education and counselling

Patient counselling was incorporated into PGx services across community, primary care, rural, and institutional settings. Pharmacists provided pre-test counselling, explained the purpose and limitations of genetic testing, interpreted results, discussed medication implications, and supported subsequent treatment decisions (Bain et al., 2019; Jeiziner et al., 2023; Liko et al., 2021; Van Heukelom et al., 2023; Wang et al., 2023). Only one study (Jeiziner et al., 2023) provided a comprehensive assessment patient-level evidence. Patients generally reported good understanding of their PGx results, increased medication knowledge, and positive perceptions of pharmacist communication and written recommendations. Sixty-two percent indicated willingness to pay for the service, suggesting perceived value. Similarly, in a different investigation (Van Heukelom et al., 2023), 77% of pre-test consultations resulted in PGx testing being ordered, indicating that pharmacist counselling supported progression from consideration of testing to informed decision-making.

These findings should nevertheless be interpreted as evidence of acceptability and patient engagement rather than clinical effectiveness. Studies (Jeiziner et al., 2023; Van Heukelom et al., 2023) discussed here involved patients already referred for PGx testing, creating selection bias toward individuals with an interest in genetic testing. In one investigation (Jeiziner et al., 2023) the same pharmacist delivered the service and conducted follow-up interviews, introducing potential social desirability bias. Across the evidence base, (Bain et al., 2019; Jeiziner et al., 2023; Liko et al., 2021; Van Heukelom et al., 2023; Wang et al., 2023) patient education outcomes were also frequently based on self-reported understanding rather than validated measures of genomic literacy or subsequent treatment behaviour.

#### Clinician education and workforce capacity

Pharmacists additionally acted as educators for physicians and other healthcare professionals. One study (Hicks et al., 2016) found that integrating pharmacist education with EHR-based PGx implementation improved guidance on test selection, interpretation, and application of recommendations. Similarly, another investigation (Van Heukelom et al., 2023) demonstrated the pharmacist’s role in supporting physicians to apply Dutch Pharmacogenetics Working Group recommendations. In oncology, Przybylski et al. (2020) reported an association between pharmacist engagement in PGx education and greater clinician comfort. These findings suggest that pharmacist education may facilitate implementation by addressing gaps in prescriber familiarity with PGx (McQuillan et al., 2026). However, the evidence does not establish the magnitude or durability of this effect. Educational outcomes were largely based on perceptions or self-reported confidence, with limited evidence of objective competency assessment or sustained changes in prescribing behaviour. Thus, pharmacist-led education appears to be an important implementation mechanism, but the literature highlights the need for standardised training and objective evaluation of PGx competency.

### Program management and health-system integration

Beyond direct clinical activities, pharmacists assumed substantial operational and leadership responsibilities within PGx programs (Liko et al., 2021). These included identifying eligible patients, coordinating testing, establishing workflows, managing result documentation, developing referral pathways, integrating PGx information into EHR systems, and coordinating communication across professional groups.

Across implementation models, pharmacists were positioned as operational coordinators, with evidence from multi-state, telehealth, and specialist clinical services demonstrating their capacity to organise testing, coordinate workflows, and integrate PGx into routine care (Bain et al., 2019; Dreischmeier et al., 2024; Van Heukelom et al., 2023). Collectively, these models suggest that the pharmacist role extends beyond post-test consultation to providing infrastructure for the delivery of PGx services. This operational role also appears feasible within pharmacy workflows, with a time-and-motion study reporting only 2.64 min of pharmacist time per simulated point-of-care (POC) encounter when preparatory and testing activities were delegated to pharmacy technicians (Bright & Klepser, 2018). However, the evidence primarily demonstrates feasibility rather than effectiveness or scalability. In particular, the simulated nature of the workflow study limits its applicability to routine practice, where competing clinical demands, interruptions, and variability in patient presentations may increase the resources required for PGx delivery.

Despite positive reports, several system-level constraints were recurrent across implementation models, including incomplete EHR interoperability, limited visibility of PGx information at prescribing, variable CDS functionality, reimbursement limitations, testing turnaround times, and fragmented communication between laboratories, pharmacists, and prescribers (Bain et al., 2018; Przybylski et al., 2020). These barriers suggest that pharmacists may be well positioned to identify and manage workflow-level challenges, but their capacity to address broader structural limitations is constrained by organisational and technological dependencies. In particular, restricted access to clinical data, limited EMR visibility, and resource constraints may impede implementation even where pharmacist-led services are established (Bain et al., 2018; Przybylski et al., 2020). Consequently, pharmacist involvement alone is unlikely to overcome barriers related to reimbursement, EHR architecture, or laboratory capacity. Effective implementation therefore appears to require coordinated organisational, informatics, and financial support alongside pharmacist expertise.

The evidence also provides limited support for scalability beyond mature health systems. Most successful models were implemented in settings with established informatics infrastructure, CDC, or dedicated resources (Bain et al., 2018; Dreischmeier et al., 2024; Liko et al., 2021; Van Heukelom et al., 2023). Their transferability to smaller or less digitally mature settings therefore remains uncertain. Furthermore, the predominantly descriptive nature of these studies limits conclusions regarding the long-term sustainability and cost-effectiveness of pharmacist-led PGx models.

### PGx research, evidence generation, and emerging technologies

A final theme concerned pharmacists’ contribution to PGx knowledge generation and the development of technologies supporting clinical implementation. Pharmacists contributed to observational analyses of DG and DDGI interactions, implementation evaluation, guideline application, and the development or assessment of computational decision-support tools. These activities rely on established PGx knowledge resources, including the PGx Knowledgebase (PharmGKB) (Gong et al., 2021) which integrates curated evidence on gene–drug relationships, clinical annotations, and prescribing information; PharmVar (Gaedigk et al., 2017), which provides standardised nomenclature and information on pharmacogene allelic variation; and the Clinical Pharmacogenetics Implementation Consortium (CPIC) (Caudle et al., 2014) which translates PGx evidence into peer-reviewed gene–drug prescribing recommendations. To grow these databases and improve knowledge, anonymized patient data collected by pharmacists is proposed. Concomitantly, pharmacist-supported PGx assessment has demonstrated the potential to identify clinically relevant medication risks, including interaction burdens and both current and prospective risks associated with pharmacotherapy (Bain et al., 2019; Chaitesipaseut et al., 2022)

Collectively, these findings support the value of pharmacists in translating PGx information into medication management, particularly in populations with complex or changing medication regimens and improving existing knowledge databases. However, the evidence remains largely focused on the identification of potential risks rather than demonstrating whether their detection leads to improved clinical outcomes, such as reduced adverse drug events, hospitalization, or healthcare expenditure (Bain et al., 2019; Chaitesipaseut et al., 2022). This limits the extent to which PGx-informed pharmacist interventions can currently be considered clinically or economically effective.

Emerging evidence also suggests that this role may be augmented by artificial intelligence (AI). A retrieval-augmented GPT-4 system incorporating CPIC guidelines and other PGx resources demonstrated stronger performance than ChatGPT 3.5 on provider-focused PGx questions, with pharmacists contributing to knowledge-base development and expert assessment (Murugan et al., 2024). While this indicates potential for AI to support access to complex PGx information, the evaluation of question-answering performance does not establish clinical reliability or safety in real-world prescribing. AI therefore appears better positioned as an adjunct to pharmacist expertise than as a substitute for clinical interpretation, particularly given the potential consequences of inaccurate or contextually inappropriate PGx recommendations.

However, the evidential basis remains limited. In research examining AI use (Przybylski et al., 2020), evaluation was restricted to a small panel of four experts, using question-based assessments rather than patient-level assessment. Moreover, performance in real clinical workflows or its effects on prescribing, patient outcomes, or medication safety were not evaluated. Additionally, superior performance on structured knowledge questions does not establish the reliability, interpretability, or clinical appropriateness of AI-generated PGx recommendations. Thus, the study (Murugan et al., 2024) provides preliminary evidence of technical feasibility and a potential informatics role for pharmacists, but does not yet support conclusions regarding clinical effectiveness, implementation readiness, or the safety of integrating such systems into routine PGx care.

Despite these limitations, across the research evidence, pharmacists appear increasingly positioned not only as users of PGx knowledge but also as contributors to its generation, curation, and translation into clinical decision-support systems. Nevertheless, the evidence remains predominantly exploratory. Small samples, retrospective data, simulated environments, expert assessment, and absence of external validation limit the generalisability of these findings. In particular, there is little evidence linking pharmacist participation in PGx research or technology development to measurable patient-level outcomes.

### PGx MTM overarching model and pharmacist roles

The literature evaluated in this review indicated that pharmacists have a multifaceted role in PGx implementation, extending from patient identification and pre-test counselling to interpretation of PGx results, medication optimization, CDC, and longitudinal follow-up. Across the included studies, pharmacists contributed to the integration of PGx information into MTM, supported the identification and resolution of DG and DDGI interactions, and translated PGx findings into actionable prescribing recommendations in collaboration with other healthcare professionals. Pharmacists also have a role in patient and clinician education, testing coordination, workflow development, and implementation of PGx services within EHR and clinical decision-support systems. Emerging models further demonstrated pharmacist involvement in programme leadership, workforce development, and the integration of PGx processes into scalable health-system workflows. Based on the critical assessment of current evidence and evaluation of implementation models, Figure 2 illustrates a potential pharmacist-led PGx-MTM pathway for pharmacist role integration in PGx programs, encompassing patient screening and counselling, testing coordination, result interpretation and clinical integration, interprofessional communication, medication optimization, and follow-up.

**Figure 2.**
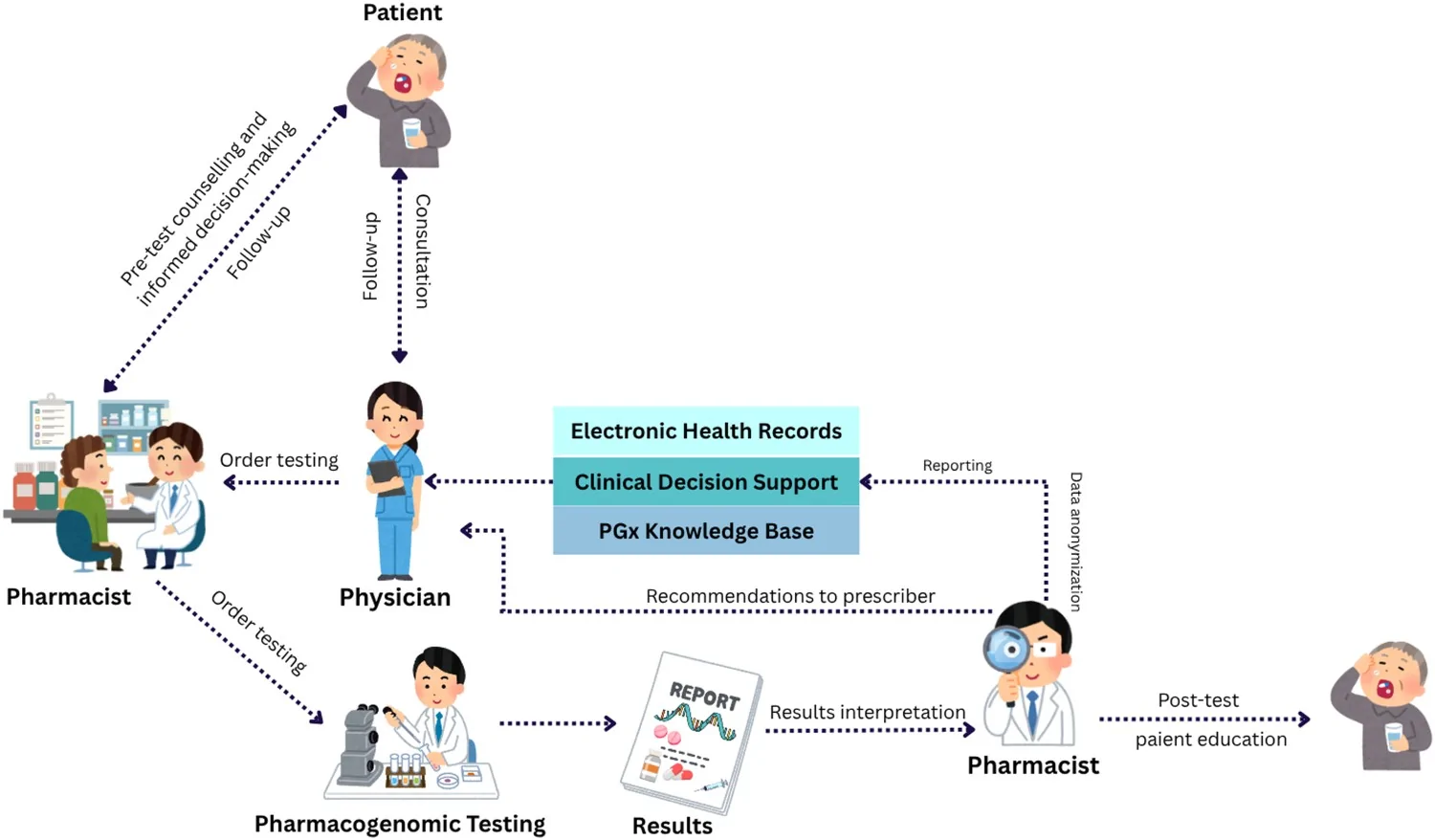
Operational workflow of the pharmacist-led PGx integration and MTM pathway.

The clinical workflow initiates with a patient presenting for clinical consultation with a physician or interacting with a clinical pharmacist due to therapeutic failure or adverse drug events. During this initial phase, the pharmacist conducts pre-test counselling and informed decision-making with the patient to outline the utility and scope of genetic testing. Following this educational intervention, PGx testing may be triggered through a synchronised process where both the physician and pharmacist possess the clinical pathway authority to order testing. Once testing is executed, laboratory data is synthesised into standardised results reports. This diagnostic information is systematically integrated into the health infrastructure via a centralised PGx knowledge base, CDS mechanisms, and EHR. To protect patient privacy while refining institutional knowledge, the workflow utilises data anonymization pathways during subsequent reporting back into the CDS ecosystem. The finalised clinical data undergoes specialised results Interpretation by the pharmacist, who translates complex genomic variants into actionable, evidence-based recommendations to the prescriber. This interprofessional communication enables the physician to safely optimise the patient’s drug regimen. The pathway culminates in targeted post-test patient education delivered by the pharmacist to improve therapeutic literacy, concluding with an ongoing loop of longitudinal clinical Follow-up shared between the physician and pharmacist to ensure long-term drug safety and efficacy.

## Discussion

This scoping review synthesised evidence on pharmacist-led PGx implementation across healthcare systems, focusing on (i) roles and responsibilities, (ii) impact on patient care and outcomes, and (iii) barriers and facilitators to integration. Across included studies, pharmacists consistently function as operational and clinical anchors of PGx services, yet the evidence base remains dominated by descriptive implementation studies with limited methodological strength and restricted linkage to long-term clinical effectiveness.

### Roles and responsibilities of pharmacists in PGx implementation

The findings suggest that pharmacists occupy a uniquely broad implementation role in PGx, extending beyond traditional medication management to encompass service delivery, clinical interpretation, informatics, education, and implementation leadership. While pharmacists are consistently identified as well positioned to support PGx adoption, the evidence indicates that their contribution is not attributable solely to pharmacotherapy expertise. Rather, their value appears to derive from their capacity to connect otherwise fragmented components of the implementation pathway, translating PGx information into clinically actionable medication decisions (Alhaddad et al., 2022; Al Maruf & Aziz, 2023; Gammal et al., 2022; Rutkowska et al., 2026; Shue et al., 2024).

This review found that pharmacists frequently coordinate testing, interpret results, support prescribing decisions, and facilitate communication between stakeholders. These activities are unlikely to be effective when considered in isolation; instead, successful PGx implementation appears to depend on their integration within a coherent workflow linking laboratory information, CDC, and prescribing (Al Maruf & Aziz, 2023; Kabbani et al., 2023; Rutkowska et al., 2026; White et al., 2022). This may help explain why implementation models that embed pharmacists across the care pathway appear more capable of translating PGx testing into clinical action than models in which pharmacists are involved only after results become available (Kabbani et al., 2023; Liang et al., 2025; Qureshi et al., 2022). However, the evidence remains insufficient to determine whether greater pharmacist integration consistently results in higher implementation uptake or improved patient outcomes.

Importantly, the findings challenge the assumption that pharmacist involvement alone is sufficient for successful implementation. Although pharmacists can function as implementation champions, persistent barriers including limited reimbursement, inadequate informatics infrastructure, fragmented EHRs, and variable clinician preparedness continue to constrain PGx adoption (Alchakee et al., 2022; Gammal & Fieg, 2022; Kabbani et al., 2023; Liang et al., 2025; Stratton & Olson, 2023). Pharmacists may therefore mitigate workflow and knowledge-related barriers but have limited influence over structural determinants such as financing, EHR architecture, and organisational capacity. Consequently, pharmacist-led models may be important for implementation but are unlikely to be sufficient without broader organisational, technological, and policy-level support.

The findings also reflect a wider shift from viewing pharmacists primarily as providers of PGx recommendations towards recognizing them as implementation specialists responsible for translating genomic evidence into routine clinical practice (Abad-Santos et al., 2024; Amaro-Álvarez et al., 2024; Cohen et al., 2013; White et al., 2022). This distinction is important because it reframes pharmacist contributions from a predominantly clinical function to one that can influence the integration, sustainability, and normalization of PGx within healthcare systems. Nevertheless, the extent to which this broader implementation role translates into sustained system-level change remains insufficiently established.

The findings suggest that pharmacist involvement is associated with improvements in medication-related processes primarily through improvements in medication-related processes rather than consistently demonstrated clinical outcomes. Across the evidence base, pharmacists contributed to prescribing optimization, identification of actionable gene–drug interactions, implementation of PGx recommendations, and patient engagement with treatment (Bain et al., 2018; Chaitesipaseut et al., 2022; Dreischmeier et al., 2024; Hicks et al., 2016; Jeiziner et al., 2023; Murugan et al., 2024; van der Wouden et al., 2020; Wang et al., 2023). However, evidence linking these process improvements to downstream clinical outcomes remains limited. Although improvements in depressive symptom scores have been reported following PGx-guided medication management in a community-based setting (Wang et al., 2023), this finding derives from a specific clinical context and cannot be assumed to represent a generalizable effect across therapeutic areas. Overall, the evidence therefore supports pharmacists as important facilitators of PGx-informed medication management, while demonstrating that the clinical impact of their involvement remains less well established than the evidence for improved implementation processes.

### Patient outcomes with pharmacist involvement

The predominance of process-oriented outcomes across the included studies is notable. Across implementation models, pharmacists were associated with improved detection of actionable variants, greater uptake of recommendations, optimization of medication therapy, and enhanced patient understanding and engagement with personalised care (Bain et al., 2018; Bright & Klepser, 2018; Hicks et al., 2016; Jeiziner et al., 2023; van der Wouden et al., 2020; Van Heukelom et al., 2023). Collectively, these findings suggest that pharmacists contribute most directly to translating PGx information into clinical decision-making, a role increasingly recognised as fundamental to effective PGx implementation (Al Maruf & Aziz, 2023; Liang et al., 2025; White et al., 2022).

However, the review also identifies a persistent evidence gap. Despite reported improvements in prescribing processes, few studies evaluated whether these changes translated into reductions in adverse drug reactions, hospitalizations, healthcare utilization, or mortality (Bain et al., 2018, 2019; Bright & Klepser, 2018; Chaitesipaseut et al., 2022; Dreischmeier et al., 2024; Hicks et al., 2016; Jeiziner et al., 2023; Murugan et al., 2024; Przybylski et al., 2020; van der Wouden et al., 2020; Van Heukelom et al., 2023; Wang et al., 2023). This is consistent with broader PGx implementation research, in which evidence of feasibility and adoption remains stronger than evidence of clinical utility and patient benefit (Abad-Santos et al., 2024; Hu et al., 2022; Shue et al., 2024; White et al., 2022). Consequently, the current evidence supports pharmacists as contributors to higher-quality PGx-informed prescribing, but remains insufficient to determine the magnitude of their effect on longer-term patient outcomes.

Importantly, this limitation may reflect the nature of the available evidence rather than the absence of clinical benefit. Most included studies were descriptive, observational, or implementation-focused and were therefore primarily designed to assess service delivery and process outcomes rather than comparative effectiveness. Pharmacist involvement consequently appears to improve care processes that are plausibly linked to better outcomes, while robust evidence demonstrating downstream clinical and economic benefits remains underdeveloped.

### Barriers and facilitators to implementation

The findings demonstrate that barriers to PGx implementation are remarkably consistent across healthcare settings, suggesting that the challenge is no longer establishing feasibility but achieving sustainable integration into routine practice. Across the evidence base, informatics limitations, organisational constraints, reimbursement challenges, and workforce-related barriers recur across care settings (Hicks et al., 2016; Przybylski et al., 2020; van der Wouden et al., 2020). This consistency indicates that implementation difficulties are rarely attributable to a single factor, but instead reflect weaknesses across interconnected components of the implementation pathway.

A notable finding is that these barriers persist even within pharmacist-led services and established implementation programmes. Difficulties with EHR integration, CDC, and interoperability of genomic information remain evident across different models of PGx delivery (Bain et al., 2019; Chaitesipaseut et al., 2022; Hicks et al., 2016). This is consistent with broader evidence indicating that technological infrastructure is not merely a facilitator of PGx delivery but a prerequisite for translating pharmacist expertise into clinical action (Liang et al., 2025; Shue et al., 2024; White et al., 2022). Without accessible and actionable genomic information at the point of prescribing, the potential contribution of pharmacist interpretation is substantially constrained.

Similarly, organisational barriers suggest that many implementation models remain dependent on local champions, institution-specific workflows, and specialised resources (Bright & Klepser, 2018; Hicks et al., 2016; Przybylski et al., 2020; van der Wouden et al., 2020). Consequently, successful programmes may demonstrate feasibility without necessarily demonstrating scalability or sustainability. This aligns with wider evidence indicating that PGx implementation remains uneven and often concentrated within settings with greater organisational and technical capacity, limiting transferability to routine practice environments (Kabbani et al., 2023; Liang et al., 2025; Virelli et al., 2021).

Importantly, the facilitators identified across the review, including pharmacist leadership, multidisciplinary collaboration, structured workflows, and integrated CDC, appear to operate synergistically rather than independently (Bright & Klepser, 2018; Hicks et al., 2016; van der Wouden et al., 2020). Their effectiveness is likely to depend on broader organisational readiness, suggesting that isolated interventions may have limited impact when supporting infrastructure is inadequate. PGx implementation should therefore be understood as a systems-level challenge requiring alignment of technological, organisational, financial, and workforce factors rather than the removal of individual barriers in isolation.

### Regulatory, legal, workforce, and reimbursement considerations

Although regulatory, legal, workforce, and reimbursement issues were not the primary focus of most included studies, they emerged as important determinants of implementation sustainability. Across the evidence, successful pharmacist-led PGx services were commonly embedded within structured healthcare systems with established governance processes, specialist expertise, and institutional support (Bain et al., 2019; Bright & Klepser, 2018; Chaitesipaseut et al., 2022; Hicks et al., 2016). This suggests that implementation success depends not only on pharmacist capability but also on the regulatory and organisational environments that determine how PGx services can be delivered and sustained.

A recurring observation across implementation models was the dependence of pharmacist roles on local scope-of-practice regulations and collaborative care arrangements (Hicks et al., 2016; Przybylski et al., 2020; van der Wouden et al., 2020; Wang et al., 2023). Pharmacists frequently interpreted PGx results and generated recommendations, while prescribing authority remained with physicians. This highlights a broader governance challenge, as inconsistent regulatory frameworks and unclear professional responsibilities may create uncertainty regarding accountability for PGx-informed decisions (Liang et al., 2025; Shue et al., 2024; White et al., 2022). As pharmacist involvement extends beyond traditional medication dispensing towards clinical interpretation and implementation leadership, the absence of clearly defined and harmonised professional responsibilities may constrain wider adoption.

Workforce capacity represents a particularly significant challenge. Although pharmacists were consistently positioned as key implementers of PGx services, relatively few studies examined whether the wider pharmacy workforce possesses the competencies required for large-scale implementation. This concern is reinforced by broader evidence demonstrating persistent gaps in PGx knowledge, confidence, and clinical preparedness among pharmacists and other healthcare professionals (Gammal et al., 2022; Kabbani et al., 2023; Smith et al., 2025; Wondrasek et al., 2024). Consequently, workforce development may represent a greater constraint on scaling PGx services than the technical capacity to perform testing itself.

Financial sustainability remains similarly unresolved. The included studies largely evaluated pilot programmes, institutionally supported services, or research-driven implementation models (Bain et al., 2018; Bain et al., 2019; Bright & Klepser, 2018; Chaitesipaseut et al., 2022; Dreischmeier et al., 2024; Hicks et al., 2016; Jeiziner et al., 2023; Murugan et al., 2024; Przybylski et al., 2020; van der Wouden et al., 2020; Van Heukelom et al., 2023; Wang et al., 2023)solated barrier than a structural determinant of whether PGx services can transition from successful pilot initiatives to sustainable routine care.

### Limitations of the evidence base and of this review

The included literature is characterised by substantial methodological heterogeneity, with predominance of cross-sectional, case series, and descriptive implementation designs. Randomised or controlled evaluations are rare. This limits causal inference regarding clinical effectiveness. Moreover, outcome reporting is inconsistent, with a strong emphasis on process metrics (e.g. test uptake, recommendation acceptance) rather than standardised clinical endpoints. Patient outcomes are fragmented and not amenable to meta-aggregation.

Additionally, publication bias likely exists, as studies disproportionately represent early adopters and well-resourced systems. This may overestimate feasibility and underrepresent implementation failure contexts. Considering these aspects, evidence in this review is restricted in terms of generalisability.

### Recommendations for practice

Applying a GRADE-informed interpretation of the evidence, practice recommendations should be differentiated according to the certainty and consistency of the available findings. The findings support continued pharmacist involvement in PGx service delivery; however, recommendations should be interpreted cautiously given the predominantly low-certainty evidence base.

Across the included studies, pharmacists consistently contributed to test coordination, result interpretation, medication optimization, and implementation of PGx recommendations (Bain et al., 2018, 2019; Bright & Klepser, 2018; Chaitesipaseut et al., 2022; Dreischmeier et al., 2024; Hicks et al., 2016; Jeiziner et al., 2023; Murugan et al., 2024; Przybylski et al., 2020; Van Heukelom et al., 2023; Wang et al., 2023; van der Wouden et al., 2020). Although evidence for direct clinical benefit remains limited, the consistency of these findings across diverse healthcare settings suggests that pharmacists are well positioned to support the integration of PGx into routine care. Accordingly, healthcare organisations implementing PGx services should consider embedding pharmacists within multidisciplinary implementation models, particularly for PGx result interpretation, medication review, and communication of recommendations to prescribers. This recommendation is supported by broader evidence highlighting the pharmacist’s role in translating genomic information into clinically actionable prescribing decisions (Al Maruf & Aziz, 2023; Liang et al., 2025; White et al., 2022).

The review also highlights a need for greater investment in workforce development. Despite recognition of pharmacists as important PGx practitioners, implementation studies continue to identify variability in clinician preparedness, knowledge, and acceptance of PGx recommendations (Przybylski et al., 2020; van der Wouden et al., 2020). Structured PGx education, continuing professional development, and interprofessional training should therefore be prioritised to improve implementation consistency and workforce readiness (Gammal et al., 2022; Kabbani et al., 2023; Smith et al., 2025; Wondrasek et al., 2024). However, the optimal educational model and its effect on sustained clinical practice remain insufficiently established.

At an organisational level, implementation efforts should prioritise integration of PGx data within EHRs and CDC systems. More developed PGx services incorporated informatics infrastructure that enabled pharmacists and prescribers to access actionable genomic information at the POC (Bain et al., 2019; Hicks et al., 2016). Wider evidence similarly identifies informatics integration as an important prerequisite for sustainable implementation (Liang et al., 2025; Shue et al., 2024; White et al., 2022). Nevertheless, investment in informatics alone is unlikely to overcome broader organisational, workforce, and governance barriers.

Finally, expansion of PGx services should occur alongside prospective evaluation of clinical, economic, and patient-centered outcomes. Given that the current evidence is dominated by feasibility and process measures rather than robust effectiveness data (Bain et al., 2019; Bright & Klepser, 2018; Chaitesipaseut et al., 2022; Dreischmeier et al., 2024; Hicks et al., 2016; Jeiziner et al., 2023; Liko et al., 2021; McQuillan et al., 2026; Murugan et al., 2024; Przybylski et al., 2020; Van Heukelom et al., 2023; van der Wouden et al., 2020; Wang et al., 2023), ongoing evaluation is necessary to determine whether improvements in prescribing quality translate into meaningful long-term health benefits and cost-effectiveness (Liang et al., 2025; White et al., 2022).

### Recommendations for future research

Future research should move beyond feasibility and descriptive implementation studies towards robust evaluative designs capable of generating higher certainty evidence. Priority should be given to controlled studies assessing clinical outcomes such as hospitalization rates, morbidity, adverse drug events, and therapy effectiveness. These outcomes remain underrepresented despite being essential for determining true clinical value. In parallel, economic evaluations are required to assess the cost-effectiveness of pharmacist-led PGx services and to inform reimbursement policy. The absence of robust pharmacoeconomic data remains a key barrier to sustained implementation in both primary and secondary care settings.

Standardization of outcome frameworks is also required to enable meaningful comparison across studies and healthcare systems. Current heterogeneity in outcome definitions significantly limits evidence synthesis and meta-analytic potential. Longitudinal real-world studies are additionally needed to assess the sustainability, scalability, and integration of PGx services into routine care pathways over time.

Finally, workforce-focused research should evaluate competency development, training effectiveness, and models of interprofessional collaboration. There is also a need for implementation science approaches that explicitly link pharmacist activities to patient-level outcomes, moving beyond process-oriented metrics to more clinically meaningful endpoints.

## Conclusion

PGx represents an important component of precision medicine, but its translation into routine care remains constrained less by scientific validity than by implementation complexity. This review demonstrates that pharmacists occupy a central role in this translation, contributing across the PGx pathway through test coordination, result interpretation, CDC, medication therapy management, patient and clinician education, and interprofessional communication. However, the current evidence remains predominantly based on feasibility studies, descriptive implementation reports, and observational designs, providing limited high-certainty evidence that pharmacist-led PGx services improve downstream clinical outcomes or reduce healthcare costs. Sustainable integration therefore requires coordinated investment in EHR integration, standardised CDC, workforce education, clearly defined pharmacist roles, and appropriate reimbursement and care pathways. As PGx advances through multi-gene testing, artificial intelligence-supported decision-making, and integrated genomic–phenotypic approaches, future research should move beyond descriptive implementation evidence towards rigorous comparative, outcome-oriented, and economic evaluations to establish whether pharmacist-led PGx implementation can translate technical feasibility into measurable and sustained improvements in medication safety, treatment effectiveness, and patient care.

## Supplementary Material

Supplementary Material _5.docx

Supplementary Material 6 (2).docx

Supplementary Material _1.docx

Supplementary Material _3.docx

Supplementary Material_4.docx

Supplementary Material _2.docx
