# Supplementary material for: Effects of pharmacists’ involvement in pharmacogenomics implementation on patient outcomes: a scoping review
Source: J Pharm Policy Pract. 2026 Nov 3;19(1):2746884. doi: 10.1080/20523211.2026.2746884 (PMC13757929; doi:10.1080/20523211.2026.2746884)
Supplement: Supplementary file 1 [file JPPP_A_2746884_SM1259.docx]

# Supplementary Material 2: Search Strategy

## Search String

("pharmacogenomics"[MeSH Terms] OR pharmacogenomic* OR pharmacogenetic* OR "precision medicine" OR "gene-drug interaction*" OR "drug-gene interaction*" OR PGx)

AND

(pharmacist* OR "clinical pharmacist*" OR "pharmacy service*" OR "pharmacist-led" OR "pharmacy practice")

AND

(implementation OR integration OR adoption OR feasibility OR workflow OR "clinical service*" OR program* OR intervention OR model OR evaluation)

AND

(healthcare OR clinic* OR hospital* OR "ambulatory care" OR "community pharmacy" OR "retirement community" OR "oncology" OR "primary care")

**Table 1**:

*Database Search*

| **Search Phrase ID** | **Integrated Search Phrase** | **Concept Coverage** | **Filter** | **Purpose / Rationale** | **Results** |
| --- | --- | --- | --- | --- | --- |
| S1 | ("pharmacogenomics"[MeSH Terms] OR "pharmacogenetics"[MeSH Terms] OR pharmacogen* OR PGx) AND (pharmacist* OR "clinical pharmacist*" OR "pharmacist-led") AND (implementation OR integration OR feasibility OR workflow OR intervention OR strategy* OR barrier* OR "patient care" OR outcome*) | PGx + pharmacist + implementation | 2015-2026  Primary Research  Books & Documents  Clinical Trials  Randomised Control Trials | Broadest integrated query capturing all three domains; high sensitivity | 15 |
| S2 | ("precision medicine"[MeSH Terms] OR "precision medicine" OR "pharmacogenomics"[MeSH Terms] OR "pharmacogenetics"[MeSH Terms] OR pharmacogen* OR PGx OR "gene-drug interaction*") AND (pharmacist* OR "clinical pharmacist*" OR "pharmacist-led" OR "pharmacy service*") AND (implementation OR adoption OR evaluation OR program* OR integration OR feasibility OR workflow OR intervention OR strategy* OR barrier* OR "patient care" OR outcome*) | Precision PGx + pharmacy practice + implementation | 2015-2026  Primary Research  Books & Documents  Clinical Trials  Randomised Control Trials | Captures structured service/program implementation studies | 25 |
| S3 | (pharmacogenomic* OR PGx) AND (pharmacist* OR "pharmacy practice") AND ("clinical service*" OR "pharmacist-led" OR model OR workflow) AND (hospital OR clinic* OR healthcare OR "primary care") | PGx + pharmacist-led services + clinical workflow + setting | 2015-2026  Primary Research  Books & Documents  Clinical Trials  Randomised Control Trials | Focus on embedded clinical service delivery models | 8 |
| S4 | ("pharmacogenomics"[MeSH Terms] OR "pharmacogenetics"[MeSH Terms] OR pharmacogen* OR PGx) AND (pharmacist* OR "clinical pharmacist*") AND (implementation OR integration OR feasibility OR strategy* OR barrier*) AND ("community pharmacy" OR "community pharmacies" OR ambulatory OR hospital* OR oncology) | PGx + pharmacist + implementation + specific healthcare settings | 2015-2026  Primary Research  Books & Documents  Clinical Trials  Randomised Control Trials | Captures real-world applied implementation contexts | 7 |
| S5 | (PGx OR pharmacogenomic* OR "drug-gene interaction*") AND ("pharmacist-led" OR pharmacist*) AND (program* OR intervention OR evaluation OR model) AND (healthcare OR clinic* OR hospital*) | PGx service programs + pharmacist leadership + evaluation | 2015-2026  Primary Research  Books & Documents  Clinical Trials  Randomised Control Trials | Focus on evaluated interventions and service models | 13 |
| S6 | ("pharmacogenomics"[MeSH Terms] OR PGx) AND ("clinical pharmacist*" OR pharmacist*) AND (implementation OR integration OR workflow OR feasibility) AND (primary care OR oncology OR "ambulatory care") | PGx + pharmacist + implementation + targeted clinical specialties | 2015-2026  Primary Research  Books & Documents  Clinical Trials  Randomised Control Trials | Captures specialty-based implementation research | 2 |

**Table 2**:

*Grey Literature Search*

| **Information Source** | **Search Approach** | **Search Terms / Strategy** | **Purpose / Rationale** | **Records Searched / Screened** |
| --- | --- | --- | --- | --- |
| Google Scholar | Advanced keyword search; results sorted by relevance; first 200 results screened per search string combination | (pharmacogenomic OR pharmacogenetic OR PGx OR "precision medicine") (pharmacist OR pharmacists OR "pharmacy services") (implementation OR integration OR feasibility OR program) (clinic OR hospital OR "primary care" OR "community pharmacy") | Identify non-indexed or early-stage evidence, including theses, reports, and non-peer-reviewed implementation studies | First 200 results per query set |
| Professional society websites (e.g., CPIC, ASHP) | Targeted manual search of publications, toolkits, implementation resources, and practice documents | pharmacogenomic implementation OR pharmacogenetic AND pharmacist-led PGx OR pharmacy practice guidelines | Capture implementation frameworks, professional guidance, and practice-oriented evidence not indexed in bibliographic databases | All relevant documents identified |
| Conference abstracts and proceedings | Manual screening of relevant pharmacogenomics and pharmacy practice conference outputs | Pharmacogen* OR PGx AND pharmacist* AND implementation OR service OR workflow | Identify emerging and unpublished implementation studies presented at scientific meetings | Relevant abstracts screened |
| Reference list screening (backward and forward citation tracking) | Manual review of bibliographies and citation tracking of included studies | Not applicable (snowballing technique) | Identify additional eligible studies missed in database and grey literature searches | All included studies screened |
