# Supplementary material for: Effects of pharmacists’ involvement in pharmacogenomics implementation on patient outcomes: a scoping review
Source: J Pharm Policy Pract. 2026 Nov 3;19(1):2746884. doi: 10.1080/20523211.2026.2746884 (PMC13757929; doi:10.1080/20523211.2026.2746884)
Supplement: Supplementary file 2 [file JPPP_A_2746884_SM1260.docx]

## Supplementary Material 4: Critical Appraisal of Selected Studies

## JBI for Cross-Sectional Studies

| **Study author: Wang et al., 2023** | | | | **Study title: Application of Pharmacogenomics**  **Testing in a Community-based Facility** | | | **Study year: 2023** | | | | | | | | |
| --- | --- | --- | --- | --- | --- | --- | --- | --- | --- | --- | --- | --- | --- | --- | --- |
|  | | | |  | | |  | | | | | | | | |
| **Internal validity** | | | | | | **Choice: comments/justification** | | **Yes** | **No** | | | **Unclear** | | **N/A** | |
| **Bias related to selection and allocation** | | | | | | | | | | | | | | | |
| **1** | **Were the criteria for inclusion in the sample clearly defined?** | | | | | Inclusion/exclusion criteria are explicitly stated. Patients with mood disorders undergoing PGx testing (2017 cohort) were identified; 87/137 met criteria after exclusions (e.g., incomplete records or missing PHQ-9 data). Selection process is clearly described, although retrospective. | | ☒ | ☒ | | | ☐ | | ☐ | |
| **2** | **Were objective, standard criteria used for measurement of the condition?** | | | | | Standardised instruments and classifications were used:   - PHQ-9 (validated depression scale) - GeneSight PGx categorisation system (structured pharmacogenomic classification) - Prescription counts and EMR timestamps (objective clinical data) | | **Yes** | **No** | | | **Unclear** | | **N/A** | |
|  | Medication gene–drug interaction classification | | | | |  |  | ☒ | ☐ | | | ☐ | | ☐ | |
|  | Provider adoption of PGx recommendations | | | | |  |  | ☒ | ☐ | | | ☐ | | ☐ | |
|  | PHQ-9 depression score | | | | |  |  | ☒ | ☐ | | | ☐ | | ☐ | |
|  | Medication count change pre/post PGx | | | | |  |  | ☒ | ☐ | | | ☐ | | ☐ | |
|  | Time delays across PGx workflow steps | | | | |  |  | ☒ | ☐ | | | ☐ | | ☐ | |
| **Bias related to classification of the exposure** | | | | | | | | | | | | | | | |
| **3** | **Was the exposure measured in a valid and reliable way?** | | | | | Exposure (PGx testing and resulting recommendations) was clearly defined using a commercial, validated PGx platform (GeneSight®). Medication exposure categories (no/moderate/significant gene–drug interaction) are standardised and reproducible. | | ☒ | ☐ | | | ☐ | | ☐ | |
| **Bias related to assessment, detection, and measurement of the outcome** | | | | | | | | | | | | | | | |
| **4** | **Were the outcomes measured in a valid and reliable way?** | | | | | Outcomes were extracted from electronic medical records and structured PGx reports. PHQ-9 is validated. However, reliance on retrospective chart abstraction introduces potential documentation variability. | | **Yes** | **No** | | | **Unclear** | | **N/A** | |
|  | Medication changes (pre/post comparison) | | | | |  |  | ☒ | ☐ | | | ☐ | | ☐ | |
|  | PHQ-9 score change | | | | |  |  | ☒ | ☐ | | | ☐ | | ☐ | |
|  | Guideline adherence (PGx consistency) | | | | |  |  | ☒ | ☐ | | | ☐ | | ☐ | |
|  | Workflow time intervals | | | | |  |  | ☒ | ☐ | | | ☐ | | ☐ | |
|  | Provider adoption of recommendations | | | | |  |  | ☒ | ☐ | | | ☐ | | ☐ | |
| **Bias related to confounding factors** | | | | | | | | | | | | | | | |
| **5** | **Were confounding factors identified?** | | | | | Authors acknowledge multiple confounders:   - Severity variability in psychiatric illness - Misclassification of diagnoses (e.g., bipolar vs depression) - Insurance constraints influencing PGx uptake - Missing follow-up data - Patient adherence and external psychosocial factors | | ☒ | ☐ | | | ☐ | | ☐ | |
| **6** | **Were strategies to deal with confounding factors stated?** | | | | | No formal adjustment (e.g., multivariate modelling, matching, or stratification) was performed. Analysis relied primarily on pre–post comparisons and descriptive statistics, with limited control of confounding. | | **Yes** | | **No** | | **Unclear** | | **N/A** | |
|  |  | | | | |  | | ☐ | | ☐ | | ☒ | | ☐ | |
|  | **Statistical conclusion validity** | | | | | | |  |  | | |  | |  | |
| **7** | **Was appropriate statistical analysis used?** | | | | |  | |  | | | | | | | |
|  |  | | | | | Appropriate tests were applied:   - Wilcoxon test for non-parametric comparisons - Paired t-test for PHQ-9 changes - Proportion comparisons with p-values   However:   - No multivariable adjustment - No sensitivity analyses - Potential clustering effects not addressed   Thus, statistical approach is appropriate but not robust for causal inference. | | **Yes** | **No** | | | **Unclear** | | **N/A** | |
|  |  |  |  |  |  |  |  | ☒ | ☐ | | | ☐ | | ☐ | |
|  | **Comprehensiveness of reporting** | | | | | | |  |  | | |  | |  | |
| **8** | **Were the study subjects and the setting described in detail?** | | | | | Extensive description of:   - Federally qualified health centre setting - Patient demographics - PGx workflow steps - Provider roles - Timeline and EMR processes - Testing platform (GeneSight®)   High transparency and reproducibility. | | **Yes** | | | **No** | | **Unclear** | | **N/A** |
|  |  | | | | |  | | ☒ | | | ☐ | | ☐ | | ☐ |
| **Overall appraisal:** | | **Include: ☒** | **Exclude: ☐** | | **Seek Further Info: ☐** | | | | | | | | | | |
| • **Strengths:**   - Clear retrospective design with defined cohort - Use of validated clinical and PGx classification systems - Objective EMR-derived outcomes - Multiple clinically relevant endpoints (prescribing behaviour, PHQ-9, workflow efficiency)   • **Key limitations (risk of bias):**   - No control group → limits causal inference - No adjustment for confounders - Missing data and attrition across workflow steps - Retrospective chart abstraction bias - Generalisability limited to similar community health settings | | | | | | | | | | | | | | | |

| **Study author:Przybylski et al.** | | | | **Study title: Current state assessment survey of challenges of pharmacogenomics within oncology pharmacy practice** | | | **Study year: 2020** | | | | | | | | |
| --- | --- | --- | --- | --- | --- | --- | --- | --- | --- | --- | --- | --- | --- | --- | --- |
|  | | | |  | | |  | | | | | | | | |
| **Internal validity** | | | | | | **Choice: comments/justification** | | **Yes** | **No** | | | **Unclear** | | **N/A** | |
| **Bias related to selection and allocation** | | | | | | | | | | | | | | | |
| **1** | **Were the criteria for inclusion in the sample clearly defined?** | | | | | The survey was distributed to professional colleagues and national oncology pharmacy listservs. Eligibility appears to be oncology pharmacists or pharmacists involved in oncology practice. | | ☒ | ☐ | | | ☐ | | ☐ | |
| **2** | **Were objective, standard criteria used for measurement of the condition?** | | | | | Standardised survey questions were used for all respondents. However, the primary outcome ("comfort with pharmacogenomic data") is a self-reported perception rather than an objectively validated clinical measure. | | **Yes** | **No** | | | **Unclear** | | **N/A** | |
|  | **C**omfort with pharmacogenomic data | | | | |  |  | ☒ | ☐ | | | ☐ | | ☐ | |
| **Bias related to classification of the exposure** | | | | | | | | | | | | | | | |
| **3** | **Was the exposure measured in a valid and reliable way?** | | | | | Exposures such as PGY2 training, years of practice, pharmacist involvement in molecular tumor boards, and institutional PGx activities appear straightforward and consistently measured through the survey instrument. | | ☒ | ☐ | | | ☐ | | ☐ | |
| **Bias related to assessment, detection, and measurement of the outcome** | | | | | | | | | | | | | | | |
| **4** | **Were the outcomes measured in a valid and reliable way?** | | | | | The study evaluated several factors potentially associated with pharmacogenomic comfort, including training, years of practice, molecular tumor board involvement, and policy development activities. These factors were recognised as potential influences on the outcome. | | **Yes** | **No** | | | **Unclear** | | **N/A** | |
|  | **C**omfort with pharmacogenomic data | | | | |  |  | ☒ | ☐ | | | ☐ | | ☐ | |
| **Bias related to confounding factors** | | | | | | | | | | | | | | | |
| **5** | **Were confounding factors identified?** | | | | | The study evaluated several factors potentially associated with pharmacogenomic comfort, including training, years of practice, molecular tumor board involvement, and policy development activities. These factors were recognised as potential influences on the outcome. | | ☒ | ☐ | | | ☐ | | ☐ | |
| **6** | **Were strategies to deal with confounding factors stated?** | | | | | Analysis was conducted using Pearson chi-square tests. No multivariable modelling, adjustment, stratification, or matching is mentioned. Potential confounding was therefore not formally addressed. | | **Yes** | | **No** | | **Unclear** | | **N/A** | |
|  |  | | | | |  | | ☐ | | ☐ | | ☒ | | ☐ | |
|  | **Statistical conclusion validity** | | | | | | |  |  | | |  | |  | |
| **7** | **Was appropriate statistical analysis used?** | | | | |  | |  | | | | | | | |
|  |  | | | | | Pearson chi-square testing is appropriate for examining associations between categorical survey variables and respondent comfort levels. However, absence of multivariable analysis limits assessment of independent predictors. | | **Yes** | **No** | | | **Unclear** | | **N/A** | |
|  |  |  |  |  |  |  |  | ☒ | ☐ | | | ☐ | | ☐ | |
|  | **Comprehensiveness of reporting** | | | | | | |  |  | | |  | |  | |
| **8** | **Were the study subjects and the setting described in detail?** | | | | | The abstract reports 84 participants from 58 cancer centres and provides information regarding respondent training and institutional characteristics. The setting is reasonably described for interpretation of findings. | | **Yes** | | | **No** | | **Unclear** | | **N/A** |
|  |  | | | | |  | | ☒ | | | ☐ | | ☐ | | ☐ |
| **Overall appraisal:** | | **Include: ☒** | **Exclude: ☐** | | **Seek Further Info: ☐** | | | | | | | | | | |
| This study is a quantitative cross-sectional survey examining barriers to implementation of pharmacogenomics services in oncology practice and factors associated with pharmacists' comfort in interpreting pharmacogenomic information. The analytical cross-sectional design is appropriate for the study objectives.  The study benefits from inclusion of respondents across 58 cancer centres, enhancing diversity of institutional perspectives. Exposures and outcomes were measured consistently across participants, and appropriate statistical analyses (Pearson chi-square tests) were used to explore associations between pharmacist characteristics and pharmacogenomic comfort levels.  The principal methodological limitations relate to potential selection bias and residual confounding. Participant recruitment through professional networks and oncology pharmacy listservs constitutes a convenience sample. Outcomes were self-reported and may be influenced by reporting bias. Although several relevant variables were examined, no multivariable analyses were conducted to control for confounding factors.  Overall, the study provides useful evidence regarding implementation barriers and workforce readiness for oncology pharmacogenomics programs and is suitable for inclusion in evidence synthesis addressing pharmacogenomics implementation, education, and service development. Overall risk of bias is moderate, primarily due to convenience sampling, self-reported outcomes, and lack of adjustment for confounding. | | | | | | | | | | | | | | | |

## JBI critical appraisal tool for quasi-experimental studies

| **Study Author: Bright et al** | | **Study Title:** Pharmacist-Provided Pharmacogenetic Point-of-Care Testing Consultation Service: A Time and Motion Study | | **Study Year: 2018** | | | | |
| --- | --- | --- | --- | --- | --- | --- | --- | --- |
|  | |  | |  | | | | |
| **Internal Validity** | | | **Choice - Comments/Justification** | | **Yes** | **No** | **Unclear** | **N/A** |
| **Bias related to temporal precedence** | | | | | | | | |
| **1** | **Is it clear in the study what is the “cause” and what is the “effect” (i.e. there is no confusion about which variable comes first)?** | | The intervention (PGx POCT workflow steps and staffing model) precedes measured outcomes (time per step and total encounter duration). Temporal ordering is explicit. | | ☒ | ☐ | ☐ | ☐ |
| **Bias related to selection and allocation** | | | | | | | | |
| **2** | **Was there a control group?** | | Single-group simulated workflow; no comparator arm. | | ☐ | ☒ | ☐ | ☐ |
| **Bias related to confounding factors** | | | | | | | | |
| **3** | **Were participants included in any comparisons similar?** | | Standardised patients and controlled simulation environment ensured homogeneity across encounters. | | ☒ | ☐ | ☐ | ☐ |
| **Bias related to administration of intervention/exposure** | | | | | | | | |
| **4** | **Were the participants included in any comparisons receiving similar treatment/care, other than the exposure or intervention of interest?** | | All encounters followed a standardised 7-step PGx POCT workflow; variation limited to random scenario assignment (antiplatelet choice), not structural care differences. | | ☒ | ☐ | ☐ | ☐ |

| **Bias related to assessment, detection and measurement of the outcome** | | | | | | |
| --- | --- | --- | --- | --- | --- | --- |
| **5** | **Were there multiple measurements of the outcome, both pre and post the intervention/exposure?** | • Time measurements were captured across multiple discrete workflow steps (7 sequential process stages), enabling within-encounter temporal comparisons. | **Yes** | **No** | **Unclear** | **N/A** |
|  | Click or tap here to enter text. |  | ☒ | ☐ | ☐ | ☐ |
|  |  |  |  |  |  |  |
| **6** | **Were the outcomes of participants included in any comparisons measured in the same way?** | Standardised time-tracking method (observer-based timing software) applied uniformly across all encounters. | **Yes** | **No** | **Unclear** | **N/A** |
|  | Click or tap here to enter text. |  | ☒ | ☐ | ☐ | ☐ |
|  |  |  |  |  |  |  |
| **7** | **Were outcomes measured in a reliable way?** |  | **Yes** | **No** | **Unclear** | **N/A** |
|  |  | Structured time-and-motion methodology with predefined workflow stages and trained observers increases measurement reliability. | ☒ | ☐ | ☐ | ☐ |

| **Bias related to participant retention** | | | | | | | | | | | |
| --- | --- | --- | --- | --- | --- | --- | --- | --- | --- | --- | --- |
| **8** | **Was follow-up complete and if not, were differences between groups in terms of their follow-up adequately described and analysed?** | | | | | | No longitudinal follow-up; study endpoints were immediate process times within simulated encounters. | | | | |
|  |  |  |  |  |  |  | **Yes** | **No** | | **Unclear** | **N/A** |
|  |  |  | | | | | ☐ | ☐ | ☐ | | ☒ |
|  |  |  | | | | |  |  |  | |  |
|  | **Statistical Conclusion Validity** | | | | | |  |  |  | |  |
| **9** | **Was appropriate statistical analysis used?** | | | | |  |  | | | | |
|  |  | | | | | Descriptive statistics (means, SDs, ranges) appropriately used for time-and-motion outcomes. No inferential analysis required for primary objective; approach aligns with methodological design. | **Yes** | **No** | **Unclear** | | **N/A** |
|  |  |  | | | | | ☒ | ☐ | ☐ | | ☐ |
|  | | | | | | | | | | | |
| **Overall appraisal:** | | | **Include: ☒** | **Exclude: ☐** | **Seek Further Info: ☐** | | | | | | |
| **Comments:**  **Methodologically, this is a low-risk workflow simulation study with strong internal consistency for time measurement, limited mainly by absence of a control group and real-world practice conditions. It is appropriate for implementation feasibility assessment rather than clinical effectiveness inference.** | | | | | | | | | | | |

## JBI Critical Appraisal Checklist for Case Series

Reviewer	Date_____19 September 2025__________________________

Author___Van Heukelom et al___________________________ Year_2023________ Record Number_______4__

|  | Yes | No | Unclear | Not applicable |
| --- | --- | --- | --- | --- |
| Were there clear criteria for inclusion in the case series? | ✓ | □ | □ | □ |
| Was the condition measured in a standard, reliable way for all participants included in the case series? | ✓ | □ | □ | □ |
| Were valid methods used for identification of the condition for all participants included in the case series? | ✓ | □ | □ | □ |
| Did the case series have consecutive inclusion of participants? | □ | □ | ✓ | □ |
| Did the case series have complete inclusion of participants? | □ | □ | ✓ | □ |
| Was there clear reporting of the demographics of the participants in the study? | ✓ | □ | □ | □ |
| Was there clear reporting of clinical information of the participants? | ✓ | □ | □ | □ |
| Were the outcomes or follow up results of cases clearly reported? | ✓ | □ | □ | □ |
| Was there clear reporting of the presenting site(s)/clinic(s) demographic information? | ✓ | □ | □ | □ |
| Was statistical analysis appropriate? | ✓ | □ | □ | □ |

Overall appraisal:	Include	✓	Exclude	□	Seek further info □

Comments (Including reason for exclusion)

This is a descriptive implementation case series of a pharmacogenomics (PGx) clinic reporting early service delivery outcomes across 102 patients. Inclusion criteria are implicit (patients seen in the PGx clinic during the first 11 months), but not formally predefined in methodological terms. Data collection appears consistent across participants, using standardised clinical workflows and EMR-based documentation.

Participant demographics (age, sex, referral type) and clinical exposure (PGx testing, visit structure, gene panel usage) are clearly reported. Outcomes are primarily descriptive (visit counts, testing uptake, variant prevalence, referral patterns), which aligns with case series methodology.

However, consecutive and complete inclusion cannot be definitively confirmed because:

- the manuscript does not explicitly state whether all eligible clinic patients were consecutively included
- research consent (n = 88 of 102) introduces partial cohort capture for some analyses

Statistical analysis is appropriate for a descriptive implementation study (proportions, means, counts), without inferential overreach.

Overall, this is a well-reported service evaluation case series with strong operational transparency, suitable for inclusion in evidence synthesis of implementation models in pharmacogenomics, though limited by incomplete clarity around sampling consecutiveness and completeness.

Reviewer	_______Date_____30 September 2025__________________________

Author___Chaitesipaseut et al.___________________________ Year_2021________ Record Number_______8__

|  | Yes | No | Unclear | Not applicable |
| --- | --- | --- | --- | --- |
| Were there clear criteria for inclusion in the case series? | ✓ | □ | □ | □ |
| Was the condition measured in a standard, reliable way for all participants included in the case series? | ✓ | □ | □ | □ |
| Were valid methods used for identification of the condition for all participants included in the case series? | ✓ | □ | □ | □ |
| Did the case series have consecutive inclusion of participants? | □ | □ | ✓ | □ |
| Did the case series have complete inclusion of participants? | □ | □ | ✓ | □ |
| Was there clear reporting of the demographics of the participants in the study? | ✓ | □ | □ | □ |
| Was there clear reporting of clinical information of the participants? | ✓ | □ | □ | □ |
| Were the outcomes or follow up results of cases clearly reported? | ✓ | □ | □ | □ |
| Was there clear reporting of the presenting site(s)/clinic(s) demographic information? | ✓ | □ | □ | □ |
| Was statistical analysis appropriate? | ✓ | □ | □ | □ |

Overall appraisal:	Include	✓	Exclude	□	Seek further info □

Comments (Including reason for exclusion)

This study is a prospective, single-arm feasibility case series evaluating the implementation of pharmacogenomic-guided medication therapy management (PGx-MTM) services within a retirement community. Clear inclusion criteria were reported, as residents were screened using a predefined list of high-evidence gene–drug pairs and enrolled if they were taking a qualifying medication.

Pharmacogenomic testing and interpretation were conducted using standardised procedures, including buccal swab collection, commercial laboratory analysis, and generation of a Personalised Medicine Report. These methods were applied consistently across participants, supporting reliable measurement and valid identification of pharmacogenomic findings.

Participant demographics, medication use, pharmacogenomic results, and pharmacist recommendations were reported descriptively. Outcomes were clearly presented, including the proportion of actionable gene–drug interactions identified and the resulting medication recommendations. The retirement community setting and academic pharmacy practice context were also adequately described.

However, consecutive inclusion of participants cannot be confirmed because the manuscript does not explicitly state whether all eligible residents were approached or enrolled consecutively during the recruitment period. Similarly, complete inclusion remains unclear because the total number of eligible residents screened and reasons for non-participation were not fully reported.

Statistical analysis consisted of descriptive statistics only, which is appropriate for a feasibility-focused case series with a small sample size (n = 18). No inappropriate inferential claims were made.

Overall, this is a well-conducted and transparently reported feasibility case series demonstrating the practical implementation of PGx-MTM services in a retirement community. While limitations exist regarding clarity of participant recruitment and completeness of inclusion, the study is suitable for inclusion in evidence syntheses focused on pharmacogenomic service implementation, feasibility, and medication optimisation in older adults.

Reviewer	_______Date_____2 October 2025__________________________

Author___Bain et al.___________________________ Year_2019________ Record Number_______9__

|  | Yes | No | Unclear | Not applicable |
| --- | --- | --- | --- | --- |
| Were there clear criteria for inclusion in the case series? | ✓ | □ | □ | □ |
| Was the condition measured in a standard, reliable way for all participants included in the case series? | ✓ | □ | □ | □ |
| Were valid methods used for identification of the condition for all participants included in the case series? | ✓ | □ | □ | □ |
| Did the case series have consecutive inclusion of participants? | □ | □ | ✓ | □ |
| Did the case series have complete inclusion of participants? | □ | □ | ✓ | □ |
| Was there clear reporting of the demographics of the participants in the study? | □ | □ | ✓ | □ |
| Was there clear reporting of clinical information of the participants? | ✓ | □ | □ | □ |
| Were the outcomes or follow up results of cases clearly reported? | ✓ | □ | □ | □ |
| Was there clear reporting of the presenting site(s)/clinic(s) demographic information? | ✓ | □ | □ | □ |
| Was statistical analysis appropriate? | ✓ | □ | □ | □ |

Overall appraisal:	Include	✓	Exclude	□	Seek further info □

Comments (Including reason for exclusion)

This study presents a descriptive evaluation of pharmacogenomic testing results among 100 participants enrolled in the Program of All-inclusive Care for the Elderly (PACE). The primary objective was to characterise the prevalence of genetic variants, drug–gene interactions (DGIs), drug–drug–gene interactions (DDGIs), and phenoconversions identified through a pharmacist-led pharmacogenomics service.

Pharmacogenomic variants and interactions were identified using standardised genetic testing and a clinical decision support system, supporting reliable measurement and valid ascertainment of outcomes across participants. Clinical information relevant to pharmacogenomic interpretation was clearly reported through counts and frequencies of DGIs, DDGIs, and phenoconversions.

However, the study used a convenience sample of participants from the PHARM-GENOME-PACE study. Consequently, inclusion criteria are only partially described, and it is unclear whether all eligible participants were considered for enrollment. Because convenience sampling was used, neither consecutive inclusion nor complete inclusion of eligible participants can be confirmed.

Outcomes were clearly reported using descriptive summaries of identified pharmacogenomic variants and interactions. The healthcare setting (PACE program) and pharmacist-led pharmacogenomics service were adequately described. Statistical analysis was appropriate for the descriptive aims of the study, consisting primarily of frequencies and proportions without unsupported inferential claims.

Overall, this study represents a descriptive pharmacogenomics case series that provides useful information regarding the prevalence of clinically relevant gene–drug and drug–drug–gene interactions in an older adult population. Although sampling methodology limits certainty regarding representativeness, the study is suitable for inclusion in evidence syntheses focused on pharmacogenomic service implementation, medication optimisation, and characterisation of pharmacogenomic findings in geriatric care settings.

Reviewer	_________________________________Date_____5 October 2025__________________________

Author___Bain et al.___________________________ Year_2020________ Record Number_______10__

|  | Yes | No | Unclear | Not applicable |
| --- | --- | --- | --- | --- |
| Were there clear criteria for inclusion in the case series? | ✓ | □ | □ | □ |
| Was the condition measured in a standard, reliable way for all participants included in the case series? | ✓ | □ | □ | □ |
| Were valid methods used for identification of the condition for all participants included in the case series? | ✓ | □ | □ | □ |
| Did the case series have consecutive inclusion of participants? | □ | □ | ✓ | □ |
| Did the case series have complete inclusion of participants? | □ | □ | ✓ | □ |
| Was there clear reporting of the demographics of the participants in the study? | ✓ | □ | □ | □ |
| Was there clear reporting of clinical information of the participants? | ✓ | □ | □ | □ |
| Were the outcomes or follow up results of cases clearly reported? | ✓ | □ | □ | □ |
| Was there clear reporting of the presenting site(s)/clinic(s) demographic information? | ✓ | □ | □ | □ |
| Was statistical analysis appropriate? | ✓ | □ | □ | □ |

Overall appraisal:	Include	✓	Exclude	□	Seek further info □

Comments (Including reason for exclusion)

This study describes the implementation of a pharmacist-led pharmacogenomics (PGx) service within the Program of All-Inclusive Care for the Elderly (PACE) and reports clinical and operational outcomes among 296 participants who underwent PGx testing. The study is best classified as a descriptive implementation case series.

Inclusion criteria were reasonably clear, consisting of PACE participants aged 55 years and older who met eligibility requirements and underwent PGx testing during the study period. PGx testing and interpretation procedures were standardised across participants, and drug-gene interactions were identified using validated laboratory testing and clinical decision support systems.

Participant demographics (age, sex, medication burden) and relevant clinical information were clearly reported. Outcomes were appropriately described, including implementation challenges, pharmacist recommendations, drug-gene interaction frequencies, and prescriber acceptance rates. The study setting was also well characterized, including the centralised pharmacy model serving PACE programs across multiple states.

However, the manuscript does not explicitly state whether participants were enrolled consecutively, nor does it clearly document whether all eligible participants during the study period were included. Therefore, consecutive and complete inclusion are judged as unclear.

Statistical methods consisted primarily of descriptive analyses (means, proportions, frequencies), which were appropriate for the study objectives and design. No inappropriate inferential claims were made.

Overall, this is a well-reported implementation-focused case series that provides valuable evidence regarding the feasibility and operationalisation of pharmacist-led PGx services in a large community-based elderly care program. Although limitations exist regarding clarity of participant inclusion procedures, the study is suitable for inclusion in evidence synthesis addressing pharmacogenomics service implementation and clinical integration.

Reviewer	_________________________________Date_____10 October 2025__________________________

Author___Dreischmeier et al. Year_2019________ Record Number_______12__

|  | Yes | No | Unclear | Not applicable |
| --- | --- | --- | --- | --- |
| Were there clear criteria for inclusion in the case series? | ✓ | □ | □ | □ |
| Was the condition measured in a standard, reliable way for all participants included in the case series? | ✓ | □ | □ | □ |
| Were valid methods used for identification of the condition for all participants included in the case series? | ✓ | □ | □ | □ |
| Did the case series have consecutive inclusion of participants? | □ | □ | ✓ | □ |
| Did the case series have complete inclusion of participants? | □ | □ | ✓ | □ |
| Was there clear reporting of the demographics of the participants in the study? | ✓ | □ | □ | □ |
| Was there clear reporting of clinical information of the participants? | ✓ | □ | □ | □ |
| Were the outcomes or follow up results of cases clearly reported? | ✓ | □ | □ | □ |
| Was there clear reporting of the presenting site(s)/clinic(s) demographic information? | ✓ | □ | □ | □ |
| Was statistical analysis appropriate? | ✓ | □ | □ | □ |

Overall appraisal:	Include	✓	Exclude	□	Seek further info □

Comments (Including reason for exclusion)

This study describes the implementation of a clinical pharmacist practitioner-led pharmacogenomics service within a Veterans Affairs hematology/oncology clinic. The primary objective was service integration and workflow development rather than evaluation of comparative clinical effectiveness. The report clearly describes the implementation process, pharmacist responsibilities, testing procedures, documentation pathways, and integration into routine clinical care.

Eligibility criteria for pharmacogenomic testing were specified, including exclusion of patients with a history of liver transplantation or allogeneic hematopoietic stem cell transplantation. Standardised testing procedures were used through the national PHASER program. The clinical setting and service model are well described, supporting reproducibility of the implementation approach.

As with many implementation-focused case series, the study relies primarily on descriptive reporting and lacks a comparator group.

Overall, this article provides useful evidence regarding operationalisation and integration of pharmacogenomics services within a Veterans Affairs oncology setting and is suitable for inclusion in evidence syntheses examining pharmacogenomics service implementation models.

# PROBAST+AI Assessment

**Study:** *Empowering Personalised Pharmacogenomics with Generative AI Solutions* (Murugan et al., 2024)

**Record:** 7

### Part A. Model Development Quality Assessment

| **Domain** | **Judgment** | **Rationale** |
| --- | --- | --- |
| Participants & Data Sources | Moderate concern | Knowledge base derived from CPIC guidelines, Dutch Pharmacogenomics Working Group recommendations, FDA labelling, and review articles. Sources are authoritative but narrowly focused on statins and a limited set of PGx genes. |
| Predictors | Moderate concern | User queries, genotype information, diplotypes, phenotypes, and contextual retrieval are described, but predictor representation and preprocessing are incompletely specified. |
| Outcome | Moderate concern | Intended outcome is accurate pharmacogenomic interpretation and recommendation generation, but no formally defined reference standard outcome exists for all query types. |
| Analysis | High concern | Prompt engineering, RAG, embedding models, and guardrails are described, but there is limited reporting of development sample size, tuning procedures, reproducibility, robustness testing, and fairness evaluation. |

### Overall Development Quality

| **Assessment** | **Rating** |
| --- | --- |
| Overall Quality Concern | **-Moderate to High** |

**Reason:** Strong clinical knowledge sources and thoughtful architecture, but insufficient methodological reporting to fully evaluate development quality according to PROBAST+AI standards.

### Part B. Model Evaluation Risk of Bias Assessment

| **Domain** | **Judgment** | **Rationale** |
| --- | --- | --- |
| Participants & Data Sources | High risk | Evaluation used a curated PGx question catalog rather than real-world prospective clinical users or external patient datasets. Spectrum bias is possible. |
| Predictors | Low–Moderate risk | Queries were predefined and relevant to intended use; however, representativeness of real clinical questions is uncertain. |
| Outcome | Moderate risk | Expert Likert-scale ratings assessed accuracy, relevance, clarity, citations, and hallucination mitigation. Useful but partly subjective outcomes. |
| Analysis | High risk | No true external validation dataset, no confidence intervals, no calibration assessment, limited statistical evaluation, and evaluation relied heavily on expert scoring. Potential optimism bias remains. |

### Overall Risk of Bias

| **Assessment** | **Rating** |
| --- | --- |
| Overall Risk of Bias | **High** |

**Reason:** Evaluation resembles an internal proof-of-concept benchmark rather than an independent external validation study. PROBAST+AI generally considers lack of rigorous external validation a major source of bias in reported performance estimates.

### Applicability Assessment

| **Domain** | **Applicability** | **Rationale** |
| --- | --- | --- |
| Participants & Data Sources | Low concern | Relevant to pharmacogenomics decision support. |
| Predictors | Low concern | Genotypes, phenotypes, and drug-gene interactions match intended PGx use. |
| Outcome | Low concern | Outcome aligns with clinical interpretation and decision-support objectives. |

### Overall Applicability

| **Assessment** | **Rating** |
| --- | --- |
| Overall Applicability Concern | **Low** |

The system was specifically designed for pharmacogenomic interpretation, so applicability to that use case is good.

## MMAT Screening Questions

Reviewer	_________________________________Date_____19 September 2025__________________________

Author___Jeiziner et al.___________________________ Year_2023________ Record Number_______3__

| **Criterion** | **Rating** | **Justification** |
| --- | --- | --- |
| S1. Are there clear research questions? | ✓ Yes | Aim clearly stated: evaluation of patients’ perspectives on pharmacist-led pharmacogenetic (PGx) service (understanding, implementation, document use, knowledge gain, willingness to pay). |
| S2. Do the collected data allow to address the research questions? | ✓ Yes | Mixed qualitative and quantitative interview data directly address all study domains (Likert scales, proportions, open responses). |

### MMAT 5. Mixed Methods Appraisal

| **Criterion** | **Rating** | **Justification** |
| --- | --- | --- |
| 5.1 Is there an adequate rationale for using a mixed methods design? | ✓ Yes | Mixed methods justified to combine quantitative description (rates, scales) with qualitative explanation (patient experiences, interpretations). |
| 5.2 Are the different components effectively integrated? | ✓ Yes | Explicit convergent design; integration via joint thematic presentation and “quantitising” of qualitative data. |
| 5.3 Are outputs of integration adequately interpreted? | ✓ Yes | Meta-inferences drawn (e.g., linking understanding with education level; linking clarity with implementation rates). |
| 5.4 Are divergences and inconsistencies adequately addressed? | ⚠ Can’t tell | Variability described (e.g., physician reactions, patient understanding), but no structured analytic reconciliation of contradictions. |
| 5.5 Do components adhere to quality criteria of each tradition? | ✓ Yes | Qualitative: appropriate interviews + thematic derivation with quotations. Quantitative: appropriate descriptive statistics with complete reporting for sample. |

### MMAT Summary

| **Domain** | **Rating** |
| --- | --- |
| 5.1 Rationale | ✓ Yes |
| 5.2 Integration | ✓ Yes |
| 5.3 Interpretation | ✓ Yes |
| 5.4 Divergences | ⚠ Can’t tell |
| 5.5 Component quality | ✓ Yes |

### Methodological interpretation (MMAT-consistent)

The study demonstrates strong methodological coherence for a convergent mixed-methods design, with clear integration of qualitative and quantitative evidence and appropriate descriptive quantitative analysis. Qualitative findings are well-supported by participant quotations and systematically categorised. The main methodological limitation under MMAT criteria is the limited explicit analytical treatment of divergences between qualitative and quantitative strands, which reduces transparency in the integration process but does not fundamentally undermine validity.

## JBI Critical Appraisal Checklist for Qualitative Research

Reviewer	Date 23 September 2025

Author . van der Wouden et al	Year	2020	Record Number 6

|  | Yes | No | Unclear |
| --- | --- | --- | --- |
| 1. Is there congruity between the philosophical or theoretical positioning of the study and the methodological approach that has been adopted | ☐ | ☐ | ☒ |
| 2. Is there congruity between the research methodology and the research question or objectives? | ☒ | ☐ | ☐ |
| 3. Is there congruity between the research methodology and the methods used to collect data? | ☒ | ☐ | ☐ |
| 4. Is there congruity between the research methodology and the representation and analysis of data? | ☒ | ☐ | ☐ |
| 5. Is there congruity between the research methodology and the interpretation of results? | ☒ | ☐ | ☐ |
| 6. Is there a statement locating the researcher culturally or theoretically? | ☐ | ☐ | ☒ |
| 7. Is the influence of the researcher on the research, and vice- versa, addressed? | ☐ | ☐ | ☒ |
| 8. Are participants, and where appropriate, their voices, adequately represented? | ☐ | ☐ | ☒ |
| 9. Is the research ethical according to current criteria, and for recent studies, is there evidence of ethical approval by an appropriate body? | ☐ | ☐ | ☒ |
| 10. Do the conclusions drawn in the research report flow from the analysis, or interpretation, of the data? | ☒ | ☐ | ☐ |

Overall appraisal:	Include ☒	Exclude ☐	Seek further info ☐

Comments (Including reason for exclusion)
This is an appropriate qualitative implementation study investigating pharmacists' perspectives on pharmacogenomic panel-testing in primary care. The use of semi-structured interviews and thematic analysis is suitable for identifying implementation barriers and facilitators. The study reached thematic saturation and identified clear, actionable themes relevant to PGx implementation. Several quality domains (researcher reflexivity, philosophical stance, participant representation, ethics approval) cannot be fully assessed from the abstract and should be verified in the full text. Nevertheless, the study provides valuable qualitative evidence regarding implementation determinants and would generally be included in a systematic review of pharmacogenomic implementation.

# JBI Critical Appraisal Checklist for textual evidence: expert opinion

Reviewer	______________________________________ Date__________23 September 2025_____________________

Author_______Hicks et al ________________________________ Year___2016______ Record Number___5______

|  | Yes | No | Unclear | Not applicable |
| --- | --- | --- | --- | --- |
| 1. Is the source of the opinion clearly identified? | ✓ | □ | □ | □ |
| 2. Does the source of opinion have standing in the field of expertise? | ✓ | □ | □ | □ |
| 3. Are the interests of the relevant population the central focus of the opinion? | ✓ | □ | □ | □ |
| 4. Does the opinion demonstrate a logically defended argument to support the conclusions drawn? | ✓ | □ | □ | □ |
| 5. Is there reference to the extant literature? | ✓ | □ | □ | □ |
| 6. Is any incongruence with the literature/sources logically defended? | ✓ | □ | □ | □ |

Overall appraisal:	Include	✓	Exclude	□	Seek further info □

Comments (Including reason for exclusion)

This article is a high-quality practice insight and implementation report from a major academic health system. The authors possess recognised expertise in pharmacogenomics and provide a detailed description of developing EHR-based clinical decision support, pharmacist consultation services, and a collaborative pharmacogenomics clinic. The article is well-supported by relevant literature and presents a coherent rationale for implementation decisions.

However, the evidence level remains limited because the article is primarily descriptive rather than evaluative. There is no formal comparative design, control group, or rigorous assessment of clinical effectiveness, cost-effectiveness, or patient outcomes. Early outcome data (211 pharmacogenomic results with 17 actionable findings) provide preliminary evidence of utility but do not establish causal impact. Despite these limitations, the article provides valuable implementation evidence and should be included in reviews examining pharmacogenomic service development, implementation strategies, or pharmacist-led pharmacogenomics programs.

# JBI Critical Appraisal Checklist for textual evidence: expert opinion

Reviewer	______________________________________ Date__________1 September 2026_________________

Author_______Liko et al ________________________________ Year___2021______ Record Number___6______

|  | Yes | No | Unclear | Not applicable |
| --- | --- | --- | --- | --- |
| 1. Is the source of the opinion clearly identified? | ✓ | □ | □ | □ |
| 2. Does the source of opinion have standing in the field of expertise? | ✓ | □ | □ | □ |
| 3. Are the interests of the relevant population the central focus of the opinion? | ✓ | □ | □ | □ |
| 4. Does the opinion demonstrate a logically defended argument to support the conclusions drawn? | ✓ | □ | □ | □ |
| 5. Is there reference to the extant literature? | ✓ | □ | □ | □ |
| 6. Is any incongruence with the literature/sources logically defended? | ✓ | □ | □ | □ |

Overall appraisal:	Include	✓	Exclude	□	Seek further info □

Comments (Including reason for exclusion)

This article is a relevant and clearly described implementation report of a pharmacist-provided pharmacogenomics service within an executive health program at an academic medical centre. The authors demonstrate appropriate expertise and provide a logically structured account of service development, including pharmacist-led pre- and post-test counselling, medication review, interpretation of PGx results, therapeutic planning, and collaboration with physicians. The report is supported by relevant literature and appropriately identifies practical implementation challenges, including limited evidence for some gene–drug pairs, communication, scheduling, and reimbursement.

However, the evidence is limited by the descriptive design, absence of a comparator group, and lack of formal assessment of clinical or patient outcomes. The independent effect of pharmacist involvement cannot therefore be established. Despite these limitations, the study provides useful implementation evidence and is appropriate for inclusion in reviews of pharmacist-provided PGx services.

# .JBI Critical Appraisal Checklist for textual evidence: expert opinion

Reviewer	____________Date__________23 September 2025_____________________

Author_______McQuillan et al ________________________________ Year___2026______ Record Number___7______

|  | Yes | No | Unclear | Not applicable |
| --- | --- | --- | --- | --- |
| 1. Is the source of the opinion clearly identified? | ✓ | □ | □ | □ |
| 2. Does the source of opinion have standing in the field of expertise? | ✓ | □ | □ | □ |
| 3. Are the interests of the relevant population the central focus of the opinion? | ✓ | □ | □ | □ |
| 4. Does the opinion demonstrate a logically defended argument to support the conclusions drawn? | ✓ | □ | □ | □ |
| 5. Is there reference to the extant literature? | ✓ | □ | □ | □ |
| 6. Is any incongruence with the literature/sources logically defended? | ✓ | □ | □ | □ |

Overall appraisal:	Include	✓	Exclude	□	Seek further info □

Comments (Including reason for exclusion)

This article provides a relevant and clearly described implementation report of a clinical pharmacy technician model supporting pharmacogenomics services within the Veterans Health Administration. The authors demonstrate appropriate expertise and provide a logical account of role development, training, workflow integration, and pharmacist oversight. The report identifies practical benefits, including reduced pharmacist administrative workload and improved timeliness and reliability of PGx processes.

However, the evidence is limited by its descriptive design, small pilot workforce, absence of a comparator group, and reliance on early qualitative experience. Clinical outcomes, cost-effectiveness, and the long-term impact of the model were not formally evaluated. The specific contribution of pharmacist involvement cannot therefore be independently quantified. Despite these limitations, the article provides valuable implementation evidence regarding workforce models, delegation, and pharmacist oversight in scaling PGx services and is appropriate for inclusion.
