# Supplementary material for: Effects of pharmacists’ involvement in pharmacogenomics implementation on patient outcomes: a scoping review
Source: J Pharm Policy Pract. 2026 Nov 3;19(1):2746884. doi: 10.1080/20523211.2026.2746884 (PMC13757929; doi:10.1080/20523211.2026.2746884)
Supplement: Supplementary file 3 [file JPPP_A_2746884_SM1261.docx]

# Supplementary Material 3: Data Synthesis

**Table 1**:

*Study Characteristics*

| NR. | Author/Date | Study Aim | Research Design & Sample | Setting and Implementation | Pharmacist Role(s) | Effects of Pharmacist Involvement | Possible Limitations |
| --- | --- | --- | --- | --- | --- | --- | --- |
| 1. | Wang et al., 2023 | To examine the use of pharmacogenomic (PGx) testing in a community-based facility, assess provider adoption of PGx recommendations, evaluate changes in psychotropic medication prescribing and patient outcomes, and identify challenges and opportunities for pharmacist involvement in PGx implementation. | Retrospective chart review of 137 patients with mood disorders undergoing PGx testing; 87 patients met the inclusion criteria and were analysed. PHQ-9 outcome analysis included 59 patients. | Federally Qualified Health Centre (Crossroad Health Centre, Cincinnati, Ohio) provides integrated primary care and behavioural health services. GeneSight® Psychotropic Test was incorporated into routine care. PGx implementation involved patient identification, testing, provider review of results, medication modification, and follow-up. | Led PGx implementation within the clinic. • Conducted pre-test patient counselling. • Provided post-test result counselling. • Interpreted PGx results and made therapeutic recommendations. • Educated providers and patients on PGx and medication use. • Facilitated integration of PGx information into prescribing decisions. • Identified opportunities to improve patient follow-up and retention throughout the PGx process. | Providers adopted PGx-guided recommendations in 88.5% of patients. • Significant increase in prescribing medications with no gene–drug interactions (44.0% to 87.4%, p<0.001). • Significant reduction in medications with moderate (35.2% to 7.9%) and significant (11.2% to 2.1%) gene–drug interactions. • PHQ-9 scores significantly improved following PGx-guided therapeutic modifications (16.1 to 11.2, p<0.001). • Pharmacist involvement was identified as improving PGx workflow efficiency, supporting therapeutic decision-making, increasing patient retention, and potentially reducing delays between implementation steps. | Single-centre retrospective design limits generalisability. • Predominantly low-income population with Medicare/Medicaid coverage. • No control group for comparison. • Potential diagnostic misclassification of psychiatric conditions. • Attrition and incomplete follow-up reduced the sample size. • Clinical significance of medication category changes was not fully established. • Outcomes could not be directly attributed to pharmacist involvement because pharmacist activities were embedded within a multidisciplinary care model. |
| 2. | Bright et al., 2018 | To quantify the time required for pharmacist-provided pharmacogenetic point-of-care testing (PGx POCT) consultations in a community pharmacy setting and assess workflow feasibility for PGx implementation. | 54 simulated PGx POCT encounters involving standardised patients presenting as post-discharge acute coronary syndrome patients requiring antiplatelet therapy optimisation. Five pharmacists participated after receiving PGx-specific training. | Simulated community pharmacy consultation rooms equipped with the Spartan RX PGx analyser. PGx testing focused on CYP2C19-guided antiplatelet therapy management. The workflow included patient assessment, sample collection, POCT testing, result interpretation, documentation, provider communication, and patient counselling. Encounters were conducted under either a traditional medication therapy management (MTM) model or a collaborative practice agreement model. | Assessed patient eligibility for PGx testing. • Obtained informed consent and answered patient questions. • Collected and managed buccal swab samples. • Interpreted CYP2C19 PGx test results. • Applied PGx findings to antiplatelet therapy recommendations. • Communicated recommendations to prescribers or implemented changes under collaborative practice agreements. • Documented results and provided patient counselling regarding PGx findings and treatment implications. • Supervised and delegated appropriate technical tasks to trained pharmacy technicians. | Demonstrated that PGx POCT can be integrated into community pharmacy workflow with a mean staff hands-on time of 9.49 minutes per encounter. • Pharmacist-specific time was only 2.64 minutes when trained pharmacy technicians performed preparatory and testing tasks. • Supported the feasibility of pharmacist-led PGx testing in community pharmacies. • Showed that collaborative practice agreements may facilitate more efficient implementation by reducing delays associated with prescriber approval. • Suggested pharmacists can provide same-day PGx-guided therapeutic recommendations, improving the timeliness of precision medicine interventions. | Simulated rather than real-world practice environment, limiting external validity. • Workflow disruptions and competing demands typical of community pharmacies were not captured. • Study evaluated workflow efficiency rather than clinical outcomes or patient health benefits. • The accuracy of pharmacists’ PGx interpretation and recommendations was not assessed. • Pharmacy technician involvement was theoretical and not directly tested. • Pharmacists had limited prior experience with the PGx service, potentially affecting timing estimates. • Focused only on CYP2C19-guided antiplatelet therapy, limiting generalisability to broader PGx applications. |
| 3. | Jeiziner et al., 2023 | To evaluate patients’ perspectives on a pharmacist-led pharmacogenetic (PGx) testing and counselling service integrated into comprehensive medication reviews in a community pharmacy. | Mixed-methods study involving patients recruited into a pharmacist-led PGx service. Follow-up interview 1 (F1): 25 patients; Follow-up interview 2 (F2): 42 patients. Patients had experienced adverse drug reactions and/or therapy failure with medications known to be affected by PGx. | Community pharmacy in Basel, Switzerland. Pharmacists provided PGx panel testing covering up to 30 genes and 100 genetic variants, integrated with medication review services. Patients received individualised PGx recommendations, a traffic-light coded list of affected medications, counselling sessions, and follow-up consultations. Semi-structured interviews were conducted 1 month and ≥4 months after service delivery. | • Identified eligible patients with adverse drug reactions or therapy failure. • Conducted PGx testing and sample collection. • Interpreted PGx panel test results. • Integrated PGx findings into comprehensive medication reviews. • Developed individualised written PGx recommendations. • Provided patient counselling and education regarding PGx results and medication implications. • Communicated recommendations to physicians and supported interprofessional decision-making. • Conducted follow-up consultations to reinforce understanding and implementation. | • At least one PGx recommendation was implemented in 69% of patients. • Patients generally demonstrated good understanding of PGx results and were able to use PGx information in medication-related decisions. • Many patients reported increased medication knowledge and awareness after pharmacist counselling. • PGx recommendations were successfully shared with referring and non-referring physicians, supporting interdisciplinary care. • Patients rated pharmacist communication, counselling, and PGx documentation highly for clarity and usability. • 62% of patients indicated willingness to pay for the pharmacist-led PGx service, suggesting perceived value. • The service facilitated patient engagement in personalised pharmacotherapy and informed medication decision-making. | • Single-site study limits generalisability. • Potential social desirability bias because the same pharmacist delivered the service and conducted follow-up interviews. • A long interval between service delivery and some interviews may have introduced recall bias. • Health literacy was inferred from education level rather than measured using validated tools. • Participants were selected from patients already referred for PGx testing, introducing selection bias. • Patient perspectives were evaluated, but clinical outcomes and objective health benefits were not directly assessed. • No comparison group was included. |
| 4. | Van Heukelom et al., 2023 | To describe the development, implementation, and early outcomes of a multidisciplinary pharmacogenomics (PGx) clinic designed to improve access to PGx services, clinician utilisation of PGx testing, and patient education across a large rural healthcare system spanning multiple states. | Descriptive implementation study/report. Early clinic data included 102 patients completing 131 clinic visits during the first 11 months of operation; 88 patients consented to participate in prospective outcomes research. Mean age 51 years; 75% female. | Sanford Health is a large rural healthcare system serving four U.S. states (South Dakota, North Dakota, Minnesota, and Iowa). A pharmacist–Advanced Practice Provider (APP) collaborative PGx clinic was established, offering both in-person and telehealth appointments. Services included pre-test counselling, PGx test ordering, post-test result interpretation, medication optimisation recommendations, provider consultation, and patient education. PGx testing was supported by an in-house genetics laboratory and integrated clinical decision support within the electronic medical record. | • Conducted comprehensive medication histories and medication reviews. • Provided pre-test counselling regarding PGx testing benefits, limitations, risks, and costs. • Interpreted PGx test results and drug–gene interactions. • Delivered post-test counselling and patient education. • Developed individualised medication recommendations based on PGx results and clinical factors. • Communicated recommendations to physicians and APPs through the electronic medical record. • Screened and triaged referrals to ensure appropriate PGx service utilisation. • Participated in clinician education and implementation activities across the health system. • Supported integration of PGx results into routine medication management and future prescribing decisions. | • Demonstrated successful implementation of a pharmacist-led component within a multidisciplinary PGx clinic serving a large rural population. • Clinician engagement was high, with 77% of patients referred by healthcare providers. • Pharmacists facilitated patient access to PGx services through both in-person and telehealth models across four states. • Of 64 pre-test visits, 77% resulted in PGx testing being ordered, suggesting pharmacist counselling supported informed patient decision-making regarding testing. • The clinic improved the availability of PGx expertise for providers who lacked confidence in ordering, interpreting, or applying PGx results. • Pharmacists contributed to system-wide PGx implementation through clinical decision support integration, provider education, and standardised medication review processes. • The collaborative model enabled comprehensive medication optimisation and personalised therapeutic recommendations. | • Primarily a descriptive implementation study without a comparator group. • Clinical effectiveness, patient outcomes, and economic outcomes were not formally evaluated in this report. • Early findings were based on a relatively small cohort from the first 11 months of clinic operation. • Single healthcare-system experience may limit generalisability to other settings. • Outcomes attributable specifically to pharmacist involvement could not be isolated because services were delivered collaboratively with APPs and other PGx team members. • Patient satisfaction, PGx literacy, and long-term clinical outcomes were still under investigation at the time of publication. • Potential selection bias as patients referred to the clinic may have had a greater interest in PGx than the general population. |
| 5. | Hicks et al., 2016 | To describe the implementation of pharmacist-managed pharmacogenomic (PGx) services within a large health system, including electronic health record (EHR)-based clinical decision support (CDS), pharmacist consultation services, and a collaborative ambulatory pharmacogenomics clinic. | Descriptive implementation/practice insights study. Health-system-wide implementation across >5 million patient visits and approximately 2.5 million medication orders annually. Early evaluation included 211 PGx results entered into the EHR during the first 3 months after CDS deployment (173 TPMT, 34 HLA-B57:01, 4 HLA-B15:02 results), including 17 actionable findings. | Cleveland Clinic Health System (Ohio and Florida), encompassing a tertiary-care hospital, children’s hospital, eight regional hospitals, and 90 ambulatory sites. PGx implementation focused initially on CPIC-guided gene–drug pairs: TPMT–thiopurines, HLA-B57:01–abacavir, and HLA-B15:02–carbamazepine. The program integrated PGx data into Epic EHR through 63 custom CDS alerts and rules, pharmacist-led review of PGx testing, EHR consultation services, and a pharmacist–physician geneticist ambulatory PGx clinic. | • Led development and implementation of PGx clinical services across the health system. • Designed and supported EHR-integrated PGx clinical decision support tools in collaboration with pharmacy informatics teams. • Conducted daily review of PGx test orders and results identified through EHR data mining. • Verified appropriate test selection and corrected ordering errors through direct communication with prescribers. • Interpreted PGx results and provided gene-based dosing and medication recommendations. • Delivered pharmacist-led EHR consultation services for PGx-related clinical questions. • Participated in a multidisciplinary ambulatory PGx clinic with physician geneticists. • Provided patient education, counselling, and PGx result interpretation. • Assisted clinicians in selecting appropriate PGx tests and applying results to pharmacotherapy decisions. | • Successfully implemented system-wide PGx CDS infrastructure with 63 custom EHR alerts supporting safer prescribing. • Pharmacist review enabled oversight of approximately 2,000 PGx tests annually across the health system. • During the first 3 months of implementation, 17 actionable PGx results were identified and acted upon. • All patients with actionable TPMT or HLA-B*57:01 findings avoided potentially harmful prescribing outcomes through therapy modification or avoidance of high-risk medications. • Pharmacist consultation services addressed complex PGx questions not captured by automated CDS. • The ambulatory clinic expanded access to PGx expertise, testing, result interpretation, and personalised medication recommendations. • Pharmacists played a central role in translating PGx information into clinical practice and supporting prescriber decision-making across multiple care settings. | • Descriptive implementation study without a control group or comparative evaluation. • Clinical outcomes, patient satisfaction, and cost-effectiveness were not formally assessed. • Short follow-up period for reported outcomes (first 3 months after CDS implementation). • The impact of pharmacist involvement could not be separated from broader multidisciplinary implementation efforts. • Resource-intensive processes, including manual annotation of some PGx results and pharmacist review of test orders, may limit scalability. • Findings reflect a single health-system experience and may not be generalisable to smaller institutions with fewer informatics and pharmacy resources. • Long-term effects on prescribing behaviour, adverse drug events, and healthcare utilisation were not evaluated. |
| 6. | van der Wouden et al., 2020 | To identify community pharmacists’ perceived barriers and enablers to implementing pre-emptive pharmacogenomic (PGx) panel testing in Dutch primary care using the Tailored Implementation for Chronic Diseases (TICD) framework. | Semi-structured interviews with 15 community pharmacists who had managed ≥2 patients with actionable PGx results. | Conducted within the Dutch arm of the PREPARE (U-PGx) implementation study. Pharmacists identified eligible patients initiating one of 39 medications with Dutch Pharmacogenetics Working Group (DPWG) recommendations, arranged 12-gene PGx panel testing, received results, entered findings into electronic medical records, and used clinical decision support systems to guide prescribing. | • Identified eligible patients for testing.  • Collected DNA samples and coordinated testing.  • Interpreted PGx results and applied DPWG recommendations.  • Entered PGx data into electronic medical records.  • Discussed recommendations with physicians.  • Reported results to patients and participated in follow-up care. | • Pharmacists viewed themselves as key leaders in PGx implementation.  • Discussed PGx recommendations with physicians in 77.2% of cases.  • Reported results directly to patients in 56.5% of cases.  • Believed PGx testing improved medication safety, individualised therapy, adherence, and potentially reduced hospital admissions.  • Strengthened pharmacist–patient and pharmacist–physician relationships.  • Increased pharmacists’ perceived professional value and confidence in medication optimisation. | Small qualitative sample (15 pharmacists).  Participants were early adopters with PGx experience, creating potential selection bias. Findings may not generalise to countries with different healthcare systems, reimbursement structures, pharmacist responsibilities, or IT infrastructure.  Focused only on pharmacists’ perspectives; views of physicians, patients, payers, and policymakers were not assessed.  Conducted within a research-supported implementation programme, which may not reflect routine practice conditions. |
| 7. | Murugan et al., 2024 | To evaluate a GPT-4–based generative AI assistant for interpreting pharmacogenomic (PGx) test results and supporting personalised medicine, clinical decision-making, and patient education. | Development and evaluation study. A retrieval-augmented generation (RAG) AI assistant was created using CPIC pharmacogenomic guidelines and related PGx resources. Performance was assessed using 80 PGx-related questions (47 provider-focused, 33 patient-focused) and evaluated by a panel of 4 experts (including pharmacists and physicians) using Likert-scale criteria. | Conducted in a pharmacogenomics context using statin-related genes (SLCO1B1, ABCG2, CYP2C9). The AI assistant integrated GPT-4 with a curated knowledge base containing CPIC guidelines, Dutch Pharmacogenomics Working Group recommendations, FDA labeling, and review literature. Responses were tailored to healthcare providers and patients through prompt engineering and safety guardrails. | Pharmacists contributed as PGx experts in study design, development of the knowledge base, questionnaire design, and expert evaluation. Pharmacist expertise informed guideline-based recommendations, medication-related interpretation of PGx results, and assessment of clinical relevance and safety of AI-generated responses. | The AI assistant demonstrated high effectiveness for provider-focused PGx queries (85% vs. 69% for ChatGPT 3.5), with superior accuracy, relevance, citation support, and reduced hallucinations. Pharmacist-guided PGx knowledge integration improved the quality and clinical applicability of medication-related recommendations. The tool showed potential to support pharmacists and other healthcare providers in interpreting complex PGx results and improving patient access to personalised medication information. | Proof-of-concept limited to statin pharmacogenomics and selected genes. No real patient data were used. Small expert evaluation panel (n=4). Context retrieval challenges reduced the accuracy for some genetic terminology. Responses occasionally contained complex language unsuitable for patients. Ethical, regulatory, privacy, and safety concerns remain unresolved. The AI assistant is not yet ready for widespread clinical implementation and requires further validation in real-world settings. |
| 8. | Chaitesipaseut et al., 2021 | To evaluate the feasibility of implementing pharmacogenetic-guided medication therapy management (PGx-MTM) services in a retirement community and determine whether drug therapy-related problems could be identified through pharmacist-led pharmacogenetic testing. | Prospective, single-arm feasibility study involving 18 retirement community residents taking at least one medication with a high-evidence pharmacogenetic association. | Conducted within an academic clinical pharmacy practice in a retirement community. Residents were screened for predefined gene–drug pairs. A clinical pharmacist collected buccal swab samples for pharmacogenetic testing, results were analysed by a commercial laboratory, and personalised medicine reports were generated. Pharmacogenetic findings for current and potential future medications were reviewed and communicated to healthcare providers. | Identified eligible participants. - Obtained consent and conducted PGx testing using buccal swabs. - Interpreted pharmacogenetic results and personalised medicine reports. - Performed medication therapy management incorporating PGx data. - Developed and communicated patient-specific recommendations to prescribers. - Identified actual and potential drug therapy-related problems. | Demonstrated that pharmacist-led PGx-MTM was feasible in a retirement community setting. Among 23 qualifying medication results, 30% (7/23) were categorised as requiring caution or consideration of alternative therapy. Pharmacists identified clinically relevant gene–drug interactions and recommended therapy modifications; one participant required an actual medication change (simvastatin). The approach also identified potential future medication risks, supporting proactive medication optimisation. | Small sample size (n = 18) and single-arm design without a control group. Conducted in a single retirement community, limiting generalisability. Feasibility rather than clinical effectiveness was assessed, and only one medication change resulted from recommendations. Short-term outcomes and provider acceptance of recommendations were not extensively evaluated. |
| 9. | Bain et al., 2019 | To evaluate pharmacogenomic test results and characterise drug–gene interactions (DGIs), drug–drug–gene interactions (DDGIs), and phenoconversions in elderly participants enrolled in a PACE program. | Convenience sample of 100 participants from the PHARM-GENOME-PACE study; observational genetic variant analysis using pharmacogenomic testing and a clinical decision support system (CDSS). | Program of All-inclusive Care for the Elderly (PACE); integration of pharmacogenomic testing with CDSS to assess clinically relevant DGIs, DDGIs, and enzyme phenoconversion patterns in polypharmacy-dense elderly population. | Pharmacists interpreted pharmacogenomic test results, utilised CDSS to identify DGIs and DDGIs, and supported clinicians in evaluating complex polypharmacy-related gene–drug and drug–drug–gene interactions. | Identified high burden of clinically relevant interactions: 146 genetic variants, 169 DGIs, 125 DDGIs, and 280 phenoconversion events. Demonstrated CYP2D6 and CYP3A4 as key loci of interaction burden. Pharmacist involvement enabled systematic identification and interpretation of complex interaction profiles, improving detection of clinically actionable risks in polypharmacy contexts. | Convenience sampling limits generalisability; observational design prevents causal inference regarding clinical outcomes; lack of patient-centred outcome measures (e.g., morbidity, hospitalisations); reliance on CDSS accuracy and completeness; potential selection bias within the PACE cohort; limited assessment of how pharmacist recommendations translated into prescribing changes or clinical benefit. |
| 10. | Bain et al., 2018 | To assess the feasibility of implementing a pharmacist-led pharmacogenomics (PGx) service within the Program of All-Inclusive Care for the Elderly (PACE). | Feasibility implementation study involving 296 PACE participants aged ≥55 years who underwent PGx testing as part of routine care. Data derived from service documentation, PGx results, and consultation records. | Centralised pharmacy model supporting community-based PACE centres across 21 U.S. states. Integration of PGx testing, interpretation, and clinical consultation into medication management workflows within a highly polypharmacy-affected elderly cohort. | Pharmacists led PGx test interpretation, identified drug–gene interactions (DGIs), provided prescribing recommendations, communicated results to prescribers, advocated for PGx integration into care decisions, and delivered education to clinicians regarding PGx application. | High prevalence of clinically relevant DGIs (majority of participants had >1 interaction; 73.6% exposed to multiple interactions). Pharmacist recommendations were highly accepted (89.0%), indicating strong clinical uptake. Demonstrated feasibility of pharmacist-led PGx integration and enhanced medication risk mitigation in a complex elderly population. | Implementation constrained by limited EHR interoperability, restricted access to patient clinical data, variability in prescriber feedback documentation, PGx knowledge gaps among stakeholders, and potential selection bias. Feasibility outcomes were descriptive, with limited evaluation of long-term clinical endpoints (e.g., hospitalisation, morbidity, cost-effectiveness). |
| 11. | Przybylski et al., 2019 | To identify institutional opportunities, barriers, and readiness factors for implementing oncology pharmacogenomics (PGx) services in health systems. | Cross-sectional online survey of 84 oncology pharmacy professionals from 58 cancer centres; analysis included subgroup comparisons based on self-reported PGx comfort level using chi-square testing. | Multicentre oncology settings across cancer centres; PGx information is primarily delivered via electronic medical records (EMR), with variability in institutional infrastructure, decision support, and access to pharmacogenomic data. | Pharmacists acted as survey respondents and (contextually) as clinical stakeholders involved in PGx interpretation, policy development, molecular tumour boards, and clinical decision support integration within oncology services. | Pharmacist involvement in PGx policy creation, tumour boards, and PGx education was significantly associated with higher self-reported comfort in interpreting pharmacogenomic data. Despite widespread engagement, major systemic barriers limited effective implementation, particularly EMR visibility issues and resource constraints. | Survey-based design limits causal inference; reliance on self-reported data introduces response and social desirability bias; moderate sample size may limit generalisability; potential selection bias from listserv distribution; heterogeneity across cancer centres is not fully controlled. |
| 12. | Dreischmeier et al., 2024 | To describe the implementation and evaluation of a pharmacist practitioner–led pharmacogenomics (PGx) service within a Veterans Affairs hematology/oncology outpatient clinic as part of a national pre-emptive testing program (PHASER). | Program implementation description within a clinical service; population comprised veterans enrolled in the PHASER pre-emptive PGx testing initiative, with exclusion of patients post–liver or stem cell transplant. | Ambulatory hematology/oncology clinic at the William S. Middleton Memorial Veterans Hospital (Madison, WI, USA). Integration of pre-emptive PGx testing into routine clinical workflows, including ordering, result reporting, EMR documentation, and clinical review embedded within established VA systems. | Clinical pharmacist practitioners designed and operationalised PGx workflows, coordinated patient selection and testing processes, interpreted PGx results, integrated findings into medication management, documented in the electronic medical record, and facilitated communication within the care team. | Successful embedding of PGx testing into routine oncology workflows demonstrated the feasibility of a sustainable, pharmacist-led model. Pharmacist integration improved structured medication management processes and enabled systematic incorporation of PGx data into therapeutic decision-making in oncology care. | Descriptive implementation design without comparator group; limited reporting of quantitative clinical outcomes (e.g., efficacy, adverse event reduction); setting-specific findings within the VA system may limit generalisability; potential implementation bias due to pharmacist-led development of workflows; absence of long-term cost-effectiveness or patient-reported outcomes. |
| 13. | Liko et al., 2021 | To describe the implementation of a pharmacist-provided pharmacogenomics (PGx) service within an executive health program (EHP), including the service development process, pharmacist–physician collaboration, clinical workflow, and practical challenges associated with PGx implementation. | Descriptive implementation report. No formal comparative sample or statistical outcome analysis was reported. The service was implemented in November 2018 as part of an executive health program. | Executive Health Program at the University of Colorado Hospital, an academic medical centre. A multidisciplinary team included 3 physicians, a PGx-trained pharmacist, a registered dietitian/exercise physiologist, a nurse, and 2 medical assistants. Four pre-implementation steps were undertaken: assessment of the patient population, selection of a PGx test, establishment of the visit structure, and selection of a billing model. PGx consultations consisted of two 1-hour visits involving pretest education, medication review, DNA collection, interpretation of results, development of a therapeutic plan, and follow-up consultation. | Led implementation of the PGx service within the EHP.  Conducted pretest PGx education and counselling.  Reviewed current medications and previous medication intolerances.  Collected DNA samples for genotyping.  Interpreted PGx test results.  Developed individualized PGx-guided therapeutic plans.  Discussed PGx results and therapeutic recommendations with physicians.  Prepared personalised PGx reports.  Conducted post-test counselling and reviewed results and recommendations with patients.  Collaborated with physicians to integrate PGx information into clinical decision-making. | Demonstrated that a pharmacist-provided PGx service was feasible within an executive health program.  Enabled the pharmacist to take a lead role in PGx implementation and clinical interpretation.  Facilitated multidisciplinary collaboration between pharmacists and physicians in developing personalised therapeutic plans.  Provided a structured process for incorporating PGx testing into patient care.  Expanded the use of precision medicine within an academic medical centre.  The implementation was considered successful overall, although several operational challenges were identified. | Descriptive report without a control or comparator group.  • No formal assessment of clinical outcomes, medication changes, patient outcomes, or cost-effectiveness was reported. Findings may have limited generalisability because the service was implemented in an executive health population within a single academic medical centre.  The EHP setting and patient population may differ substantially from routine primary care or other healthcare settings.  PGx evidence was limited for some gene–drug pairs, creating uncertainty in therapeutic interpretation.  Communication of PGx information to non-EHP providers was challenging.  Scheduling and reimbursement presented practical barriers to service delivery.  The study describes implementation rather than quantitatively demonstrating the independent effect of pharmacist involvement. |
| 14. | McQuillan et al., 2026 | To describe the development and early implementation of **a clinical pharmacy technician (CPhT) model** designed to support and scale pharmacogenomics (PGx) services within the Veterans Health Administration (VHA), while allowing pharmacists to focus on advanced clinical PGx care and program leadership. | Descriptive report of an early implementation/pilot programme. Six PGx CPhT positions were established in 2024 at national, regional, and local levels. The pilot was implemented across 2 regions with different operational models, with additional national-level support for targeted PGx care. The report describes early qualitative implementation experiences rather than a controlled evaluation. | Veterans Health Administration (VHA), the largest integrated health system implementing PGx, with 157 facilities offering testing and >150,000 PGx orders completed. As the National Pharmacogenomics Program (NPP) expanded, Clinical Pharmacist Practitioners (CPPs) experienced increasing operational workload. The CPhT model was developed in 2023 by national PGx leaders, regional pharmacy executives, and local PGx champions and implemented in 2024 to redistribute operational tasks while retaining pharmacist oversight of clinically complex PGx activities. | Retained responsibility for advanced PGx clinical care and programme leadership.  Designed and implemented the CPhT model in collaboration with national, regional, and local PGx stakeholders.  Provided standardised CPhT training in PGx fundamentals, communication, informatics tools, and electronic health record workflows.  Reviewed CPhT-prepared documentation and draft notes.  Provided clinical oversight of triaged gene–drug alerts.  Managed higher-risk and clinically complex PGx findings requiring pharmacist expertise.  Oversaw integration of CPhT activities into PGx workflows.  Supported delegation of patient identification, outreach, consent, laboratory coordination, and post-test triage to appropriately trained technicians. | Early qualitative experience suggested reduced administrative workload for CPPs, allowing pharmacists to concentrate on advanced PGx care and programme leadership.  • Improved the timeliness and reliability of PGx processes.  • Enabled more efficient identification of patients requiring testing and management of routine PGx workflow tasks.  • Maintained pharmacist involvement for clinically complex decisions while delegating lower-complexity operational activities.  Enhanced CPhT career development and professional advancement.  No voluntary CPhT turnover had occurred at the time of reporting.  Demonstrated a potentially scalable model for expanding PGx services within existing pharmacy resources. | Early descriptive implementation report, so long-term effectiveness has not yet been established. No control or comparator group was reported. Outcomes were primarily based on qualitative early experience, limiting quantitative assessment of impact. The independent contribution of pharmacists versus CPhTs to improvements in PGx service delivery was not formally measured. Findings may have limited generalisability because VHA is a large, integrated healthcare system with infrastructure that may not be available in other settings. Only six CPhT positions were included in the initial pilot. The two regional models may have differed in workflow and implementation, potentially limiting direct comparison. The model requires appropriate training, pharmacist oversight, informatics infrastructure, and clearly defined delegation processes, which may create implementation barriers in less-resourced systems. • Longer-term effects on clinical outcomes, patient safety, cost-effectiveness, and workforce capacity remain to be evaluated. |
