# Supplementary material for: Effects of pharmacists’ involvement in pharmacogenomics implementation on patient outcomes: a scoping review
Source: J Pharm Policy Pract. 2026 Nov 3;19(1):2746884. doi: 10.1080/20523211.2026.2746884 (PMC13757929; doi:10.1080/20523211.2026.2746884)
Supplement: Supplementary file 5 [file JPPP_A_2746884_SM1263.docx]

# Supplementary Material 6

## Thematic Analysis

| **Theme / Pharmacist Function** | **Studies Evidencing Theme** | **Core Pharmacist Activities Across Studies** | **Implementation Contexts** | **Cross-cutting Effects of Pharmacist Involvement** | **Key Barriers / Limitations** |
| --- | --- | --- | --- | --- | --- |
| **1. PGx Test Ordering, Coordination, and Workflow Integration** | Wang 2023; Bright 2018; Jeiziner 2023; Van Heukelom 2023; Chaitesipaseut 2021; Dreischmeier 2024 | Identifying eligible patients; initiating PGx testing; coordinating sample collection (buccal swabs/POCT); embedding PGx into clinical workflows; integrating testing into routine care pathways | Community pharmacies, primary care clinics, retirement communities, VA oncology clinics, rural health systems | Enabled operational feasibility of PGx services; reduced fragmentation of testing processes; supported smoother integration of precision medicine into routine care | Workflow complexity; limited interoperability of systems; variable infrastructure maturity; small-scale or site-specific implementation models |
| **2. Interpretation of PGx Results and Clinical Translation** | All studies (especially Hicks 2016; Bain 2018/2019; Wang 2023; Dreischmeier 2024) | Interpreting gene–drug interactions (DGIs), drug–drug–gene interactions (DDGIs), and guideline-based recommendations; applying CPIC/DPWG/FDA guidance; synthesizing results into actionable medication changes | Across all settings including health systems, oncology, psychiatry, VA clinics, and community pharmacies | Centralised expert interpretation improved detection of clinically actionable variants; increased identification of high-risk prescribing; enhanced precision of medication decisions | Reliance on guideline completeness; variability in PGx literacy among clinicians; dependence on clinical decision support accuracy |
| **3. Clinical Decision Support (CDS) and Informatics Integration** | Hicks 2016; Bain 2019; Van Heukelom 2023; Murugan 2024 | Development and use of EHR-integrated alerts; CDS optimisation; embedding PGx into electronic prescribing systems; leveraging AI tools for interpretation | Large integrated health systems, oncology networks, experimental AI platforms | Improved scalability of PGx interpretation; enabled system-wide alerts preventing harmful prescribing; facilitated real-time decision support | High resource demand; interoperability limitations; alert fatigue; experimental/early-stage AI validation issues |
| **4. Provider Consultation, Communication, and Interprofessional Collaboration** | Wang 2023; Jeiziner 2023; Hicks 2016; Van der Wouden 2020; Dreischmeier 2024 | Communicating PGx findings to prescribers; recommending therapy changes; participating in multidisciplinary teams (e.g., tumor boards, MTM models); liaising between labs and clinicians | Oncology clinics, primary care, community pharmacies, national implementation programs | High acceptance of pharmacist recommendations (up to 89%); strengthened physician confidence in PGx-guided prescribing; improved interprofessional trust | Variable provider engagement; inconsistent documentation of response; heterogeneity in prescriber uptake across systems |
| **5. Patient Education, Counselling, and Shared Decision-Making** | Wang 2023; Jeiziner 2023; Bright 2018; Van Heukelom 2023; Dreischmeier 2024 | Pre- and post-test counselling; explaining genetic results; supporting informed consent; improving medication literacy; guiding therapy expectations | Community pharmacies, outpatient clinics, VA clinics, primary care settings | Improved patient understanding of PGx; increased engagement and acceptance of personalized therapy; enhanced willingness to act on recommendations | Health literacy variability; recall bias; time burden on pharmacists; lack of standardized PGx education tools |
| **6. Medication Therapy Management and Clinical Optimization** | Wang 2023; Jeiziner 2023; Chaitesipaseut 2021; Hicks 2016 | Identifying drug therapy problems; recommending medication changes; optimizing psychotropic, antiplatelet, statin, and immunosuppressive therapy; proactive risk mitigation | Psychiatry, cardiology, oncology, geriatric and retirement populations | Demonstrated measurable improvements in prescribing appropriateness; reduced gene–drug interaction burden; improved symptom outcomes in some settings (e.g., PHQ-9) | Limited causal attribution; lack of control groups; inconsistent reporting of clinical endpoints |
| **7. Education, Training, and Capacity Building of Clinicians** | Przybylski 2019; Hicks 2016; Van der Wouden 2020 | Educating prescribers; developing PGx policies; improving clinician comfort; supporting PGx competency development | Oncology systems, national implementation programs, hospital systems | Increased clinician confidence and PGx uptake; association between pharmacist involvement and higher PGx competence | Persistent knowledge gaps; uneven training availability; reliance on early adopters |
| **8. Implementation Leadership and System-Level Integration** | Hicks 2016; Van Heukelom 2023; Bain 2018 | Designing PGx services; leading institutional rollout; developing workflows; standardizing protocols; embedding PGx into health system strategy | Large health systems, VA networks, rural multi-state systems | Demonstrated pharmacists as central implementation leaders; enabled scalable PGx infrastructure; supported institutionalisation of precision medicine | High implementation complexity; resource intensity; scalability constraints outside large systems |
| **9. Identification of PGx Barriers and Health System Challenges** | Przybylski 2019; van der Wouden 2020; Hicks 2016 | Identifying EMR limitations; insurance barriers; lack of resources; workflow inefficiencies; CDS visibility issues | Oncology networks, primary care systems, European PGx pilots | Provided critical insights into systemic barriers; informed future implementation strategies | Structural barriers (EMR design, reimbursement, turnaround time); heterogeneity across systems |
| **10. Evidence Generation and PGx Knowledge Development** | Bain 2019; Murugan 2024; Hicks 2016 | Characterizing DGIs/DDGIs; evaluating AI-supported PGx interpretation; generating implementation data for CPIC-guided drugs | Research-integrated clinical programs and experimental AI systems | Expanded evidence base for PGx implementation; demonstrated clinical relevance of complex interaction patterns | Limited patient outcomes; early-stage validation; reliance on simulated or observational datasets |
