# Supplementary material for: Effects of pharmacists’ involvement in pharmacogenomics implementation on patient outcomes: a scoping review
Source: J Pharm Policy Pract. 2026 Nov 3;19(1):2746884. doi: 10.1080/20523211.2026.2746884 (PMC13757929; doi:10.1080/20523211.2026.2746884)
Supplement: Supplementary file 6 [file JPPP_A_2746884_SM1264.docx]

# Supplementary Material 5: Grade Criteria Assessment

**Table 1:**

*Outcome 1: Pharmacist Roles in Pharmacogenomics Implementation*

| **Domain** | **Assessment** |
| --- | --- |
| Study designs | Case series, cross-sectional surveys, quasi-experimental implementations, expert opinion, qualitative studies |
| Risk of bias | Serious (non-randomized designs, selection bias, lack of controls, reliance on descriptive reporting) |
| Inconsistency | Moderate (roles consistent: interpretation, CDS integration, counselling, workflow design; variability in scope across settings) |
| Indirectness | Low (directly relevant to pharmacist PGx roles across specialties) |
| Imprecision | Moderate (mostly descriptive, limited effect estimates) |
| Publication bias | Likely (implementation success reporting bias probable) |

**GRADE certainty: LOW**

**Rationale:** Evidence consistently shows pharmacists function as PGx interpreters, workflow integrators, and clinical advisors, but findings are derived from non-comparative descriptive studies.

**Table 2:**

*Outcome 2: Impact of Pharmacist-led PGx Interventions on Patient Care and Clinical Outcomes*

| **Domain** | **Assessment** |
| --- | --- |
| Study designs | Mostly observational pre–post, case series, feasibility studies |
| Risk of bias | Serious to very serious (no control groups, confounding not adjusted, retrospective extraction common) |
| Inconsistency | Moderate (positive signals: medication changes, DGI resolution, PHQ-9 improvement, acceptance rates 70–90%) |
| Indirectness | Moderate (surrogate outcomes dominate: prescribing behaviour, recommendations, workflow metrics) |
| Imprecision | Serious (small samples, heterogeneous endpoints, limited statistical inference) |
| Publication bias | Likely high |

**GRADE certainty: VERY LOW to LOW (borderline LOW for process outcomes)**

**Key interpretation:**

- Strong signal for implementation effectiveness (acceptance rates, workflow feasibility)
- Weak evidence for clinical outcome improvement causality

**Table 3**:

*Outcome 3: Barriers and Facilitators to Pharmacist Integration in PGx*

| **Domain** | **Assessment** |
| --- | --- |
| Study designs | Cross-sectional surveys, qualitative interviews, implementation case series |
| Risk of bias | Moderate (self-report, convenience sampling, limited reflexivity reporting in some qualitative work) |
| Inconsistency | Low (high convergence across studies) |
| Indirectness | Low |
| Imprecision | Low–moderate (adequate thematic saturation across multiple studies) |
| Publication bias | Unclear |

**GRADE certainty: MODERATE**

**Key consistent findings:**
Facilitators:

- Pharmacist expertise in medication management
- CDS tools (EHR-integrated PGx)
- Institutional PGx programs (e.g., VA PHASER, PACE models)
- Multidisciplinary collaboration

Barriers:

- EHR fragmentation
- Lack of PGx education/training
- Insurance and reimbursement issues
- Turnaround time for testing
- Limited provider familiarity

**Table 4**:

*Outcome 4: Feasibility and Implementation of Pharmacogenomics Services*

| **Domain** | **Assessment** |
| --- | --- |
| Study designs | Case series, quasi-experimental workflow studies, implementation reports |
| Risk of bias | Serious (no controls, selection bias, implementation confounding) |
| Inconsistency | Low (high agreement that implementation is feasible) |
| Indirectness | Low |
| Imprecision | Moderate |
| Publication bias | Likely |

**GRADE certainty: LOW**

**Key finding:**
All implementation studies consistently demonstrate feasibility, but evidence is limited to descriptive service evaluations.

**Table 5**:

*Overall Evidence Profile Across All 12 Studies*

| **Outcome domain** | **Certainty (GRADE)** |
| --- | --- |
| Pharmacist roles in PGx | LOW |
| Clinical impact of pharmacist-led PGx | VERY LOW–LOW |
| Barriers & facilitators | MODERATE |
| Feasibility/implementation success | LOW |

## Summary Statement

Across heterogeneous observational, implementation, survey, and qualitative studies, pharmacist-led pharmacogenomics services demonstrate consistent feasibility and clearly defined professional roles, particularly in medication therapy optimisation, interpretation of genetic results, and integration of clinical decision support systems. However, certainty in clinical effectiveness remains low due to non-comparative study designs, small sample sizes, and substantial risk of bias. Evidence supporting barriers and facilitators to implementation is of moderate certainty due to strong convergence across multiple methodological traditions.
